# Supplementary material for: Semi-solid prodrug nanoparticles for long-acting delivery of water-soluble antiretroviral drugs within combination HIV therapies
Source: Nat Commun. 2019 Mar 29;10:1413. doi: 10.1038/s41467-019-09354-z (PMC6441007; doi:10.1038/s41467-019-09354-z)
Supplement: Supplementary file 1 — Supplementary Information [file 41467_2019_9354_MOESM1_ESM.pdf]

Semi-solid prodrug nanoparticles for long-acting delivery of water-soluble antiretroviral drugs within combination HIV therapies

James J. Hobson et al

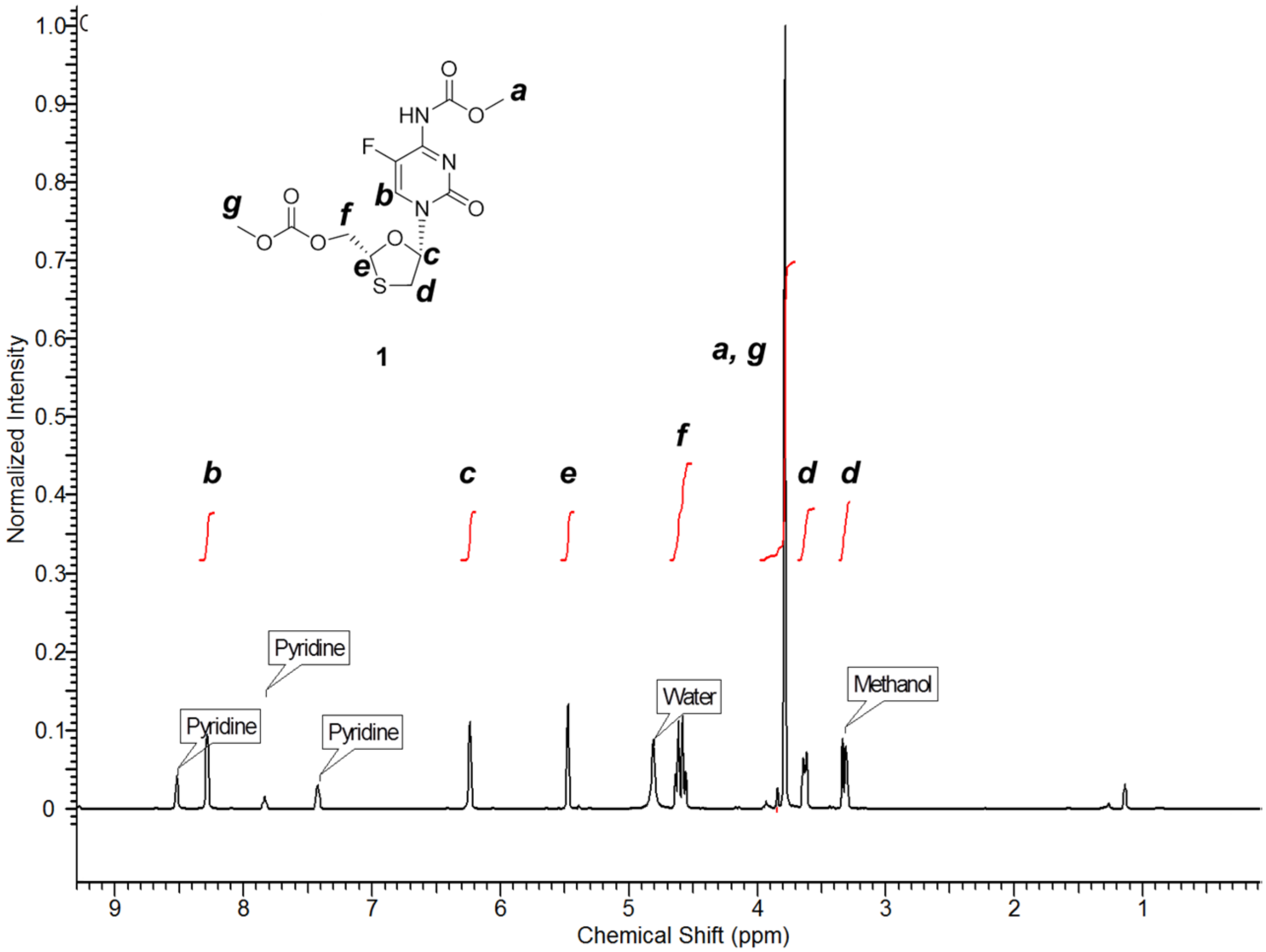

Supplementary Figure 1. ^{1}H NMR (500 MHz) of **1** in CD_3OD.

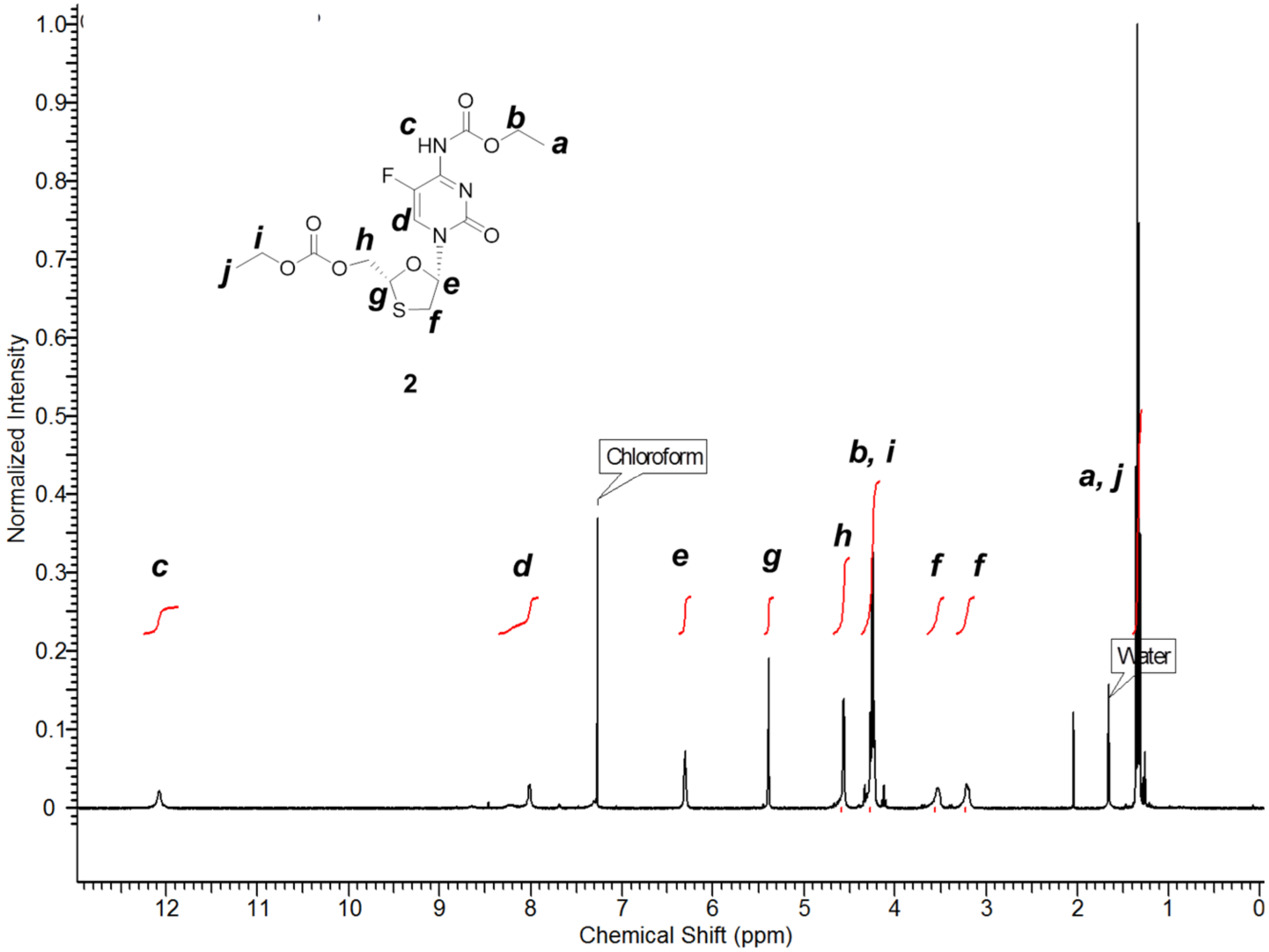

Supplementary Figure 2. ^{1}H NMR (500 MHz) of **2** in $CDCl_3$.

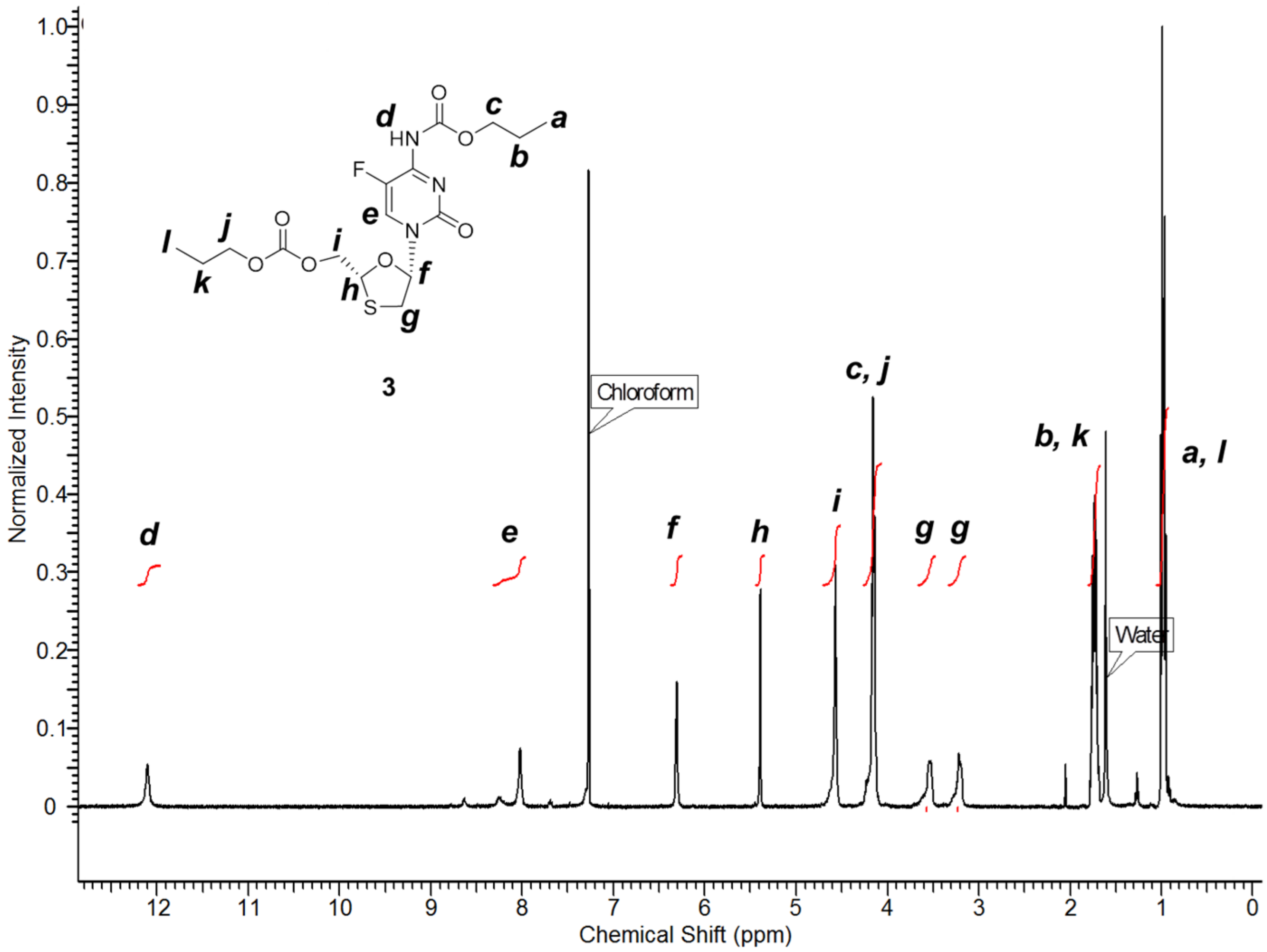

Supplementary Figure 3. ^{1}H NMR (500 MHz) of **3** in $CDCl_3$.

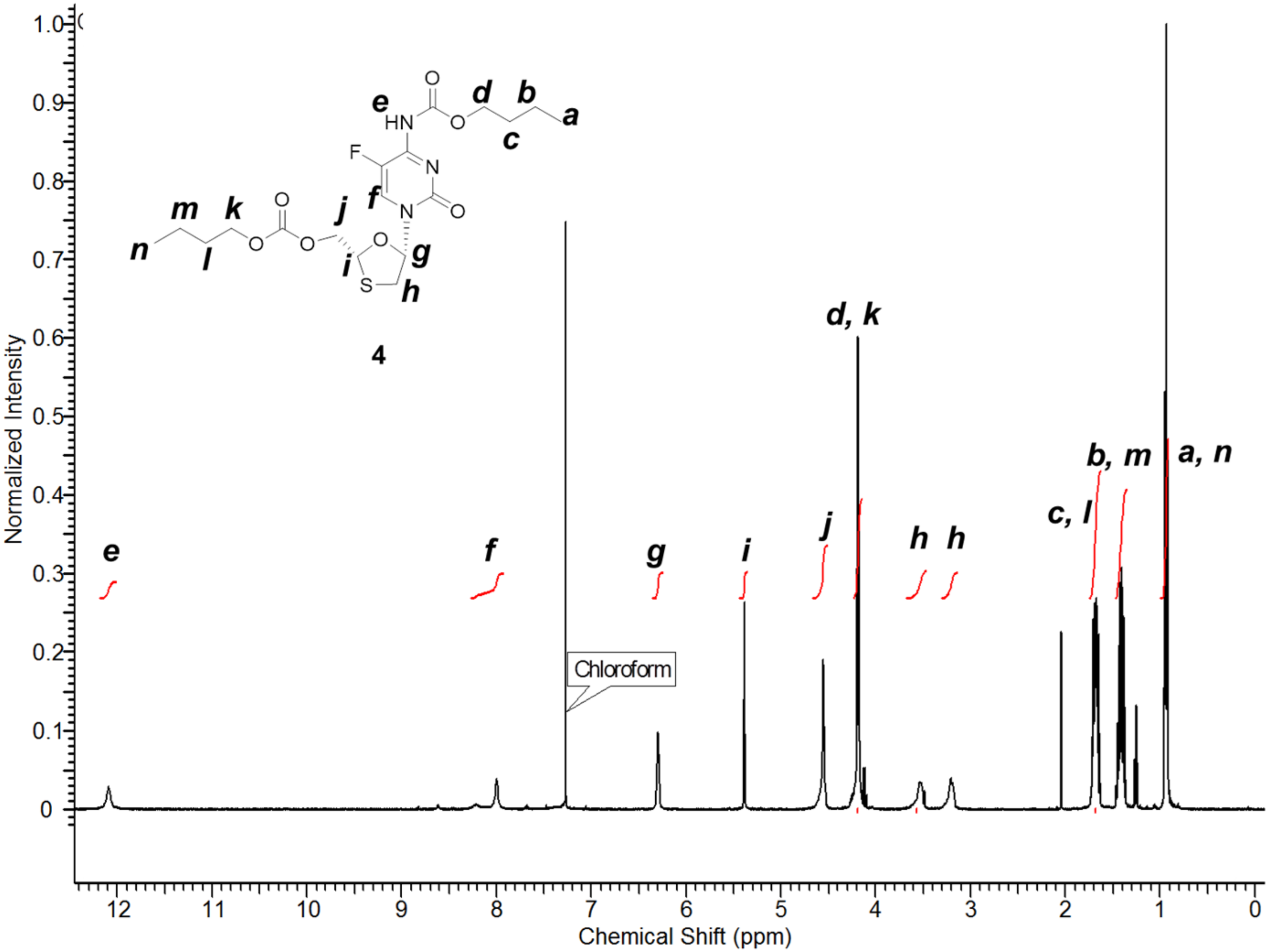

Supplementary Figure 4. ^{1}H NMR (500 MHz) of **4** in $CDCl_3$.

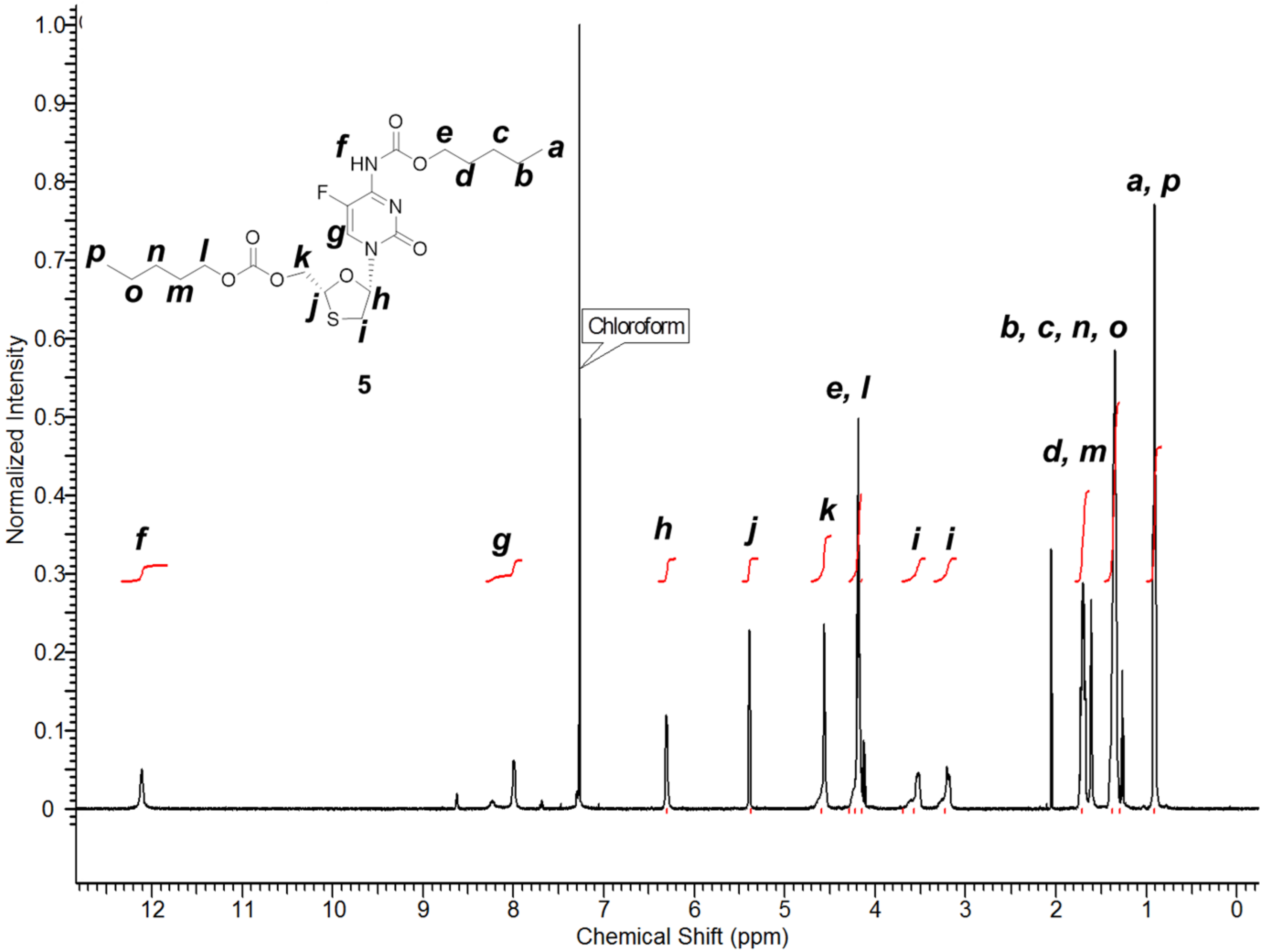

Supplementary Figure 5. ^{1}H NMR (500 MHz) of **5** in $CDCl_3$.

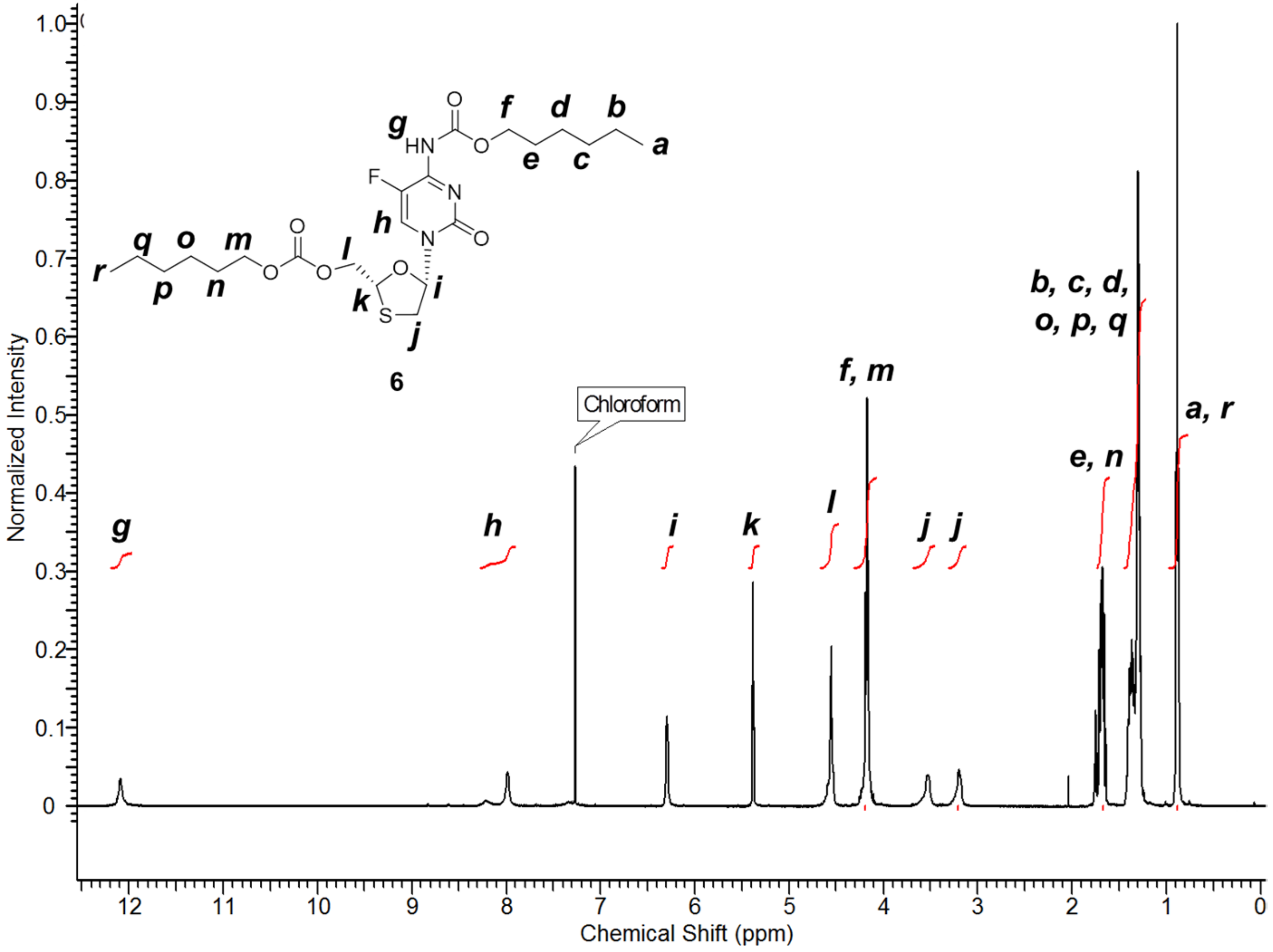

Supplementary Figure 6. ^{1}H NMR (500 MHz) of **6** in $CDCl_3$.

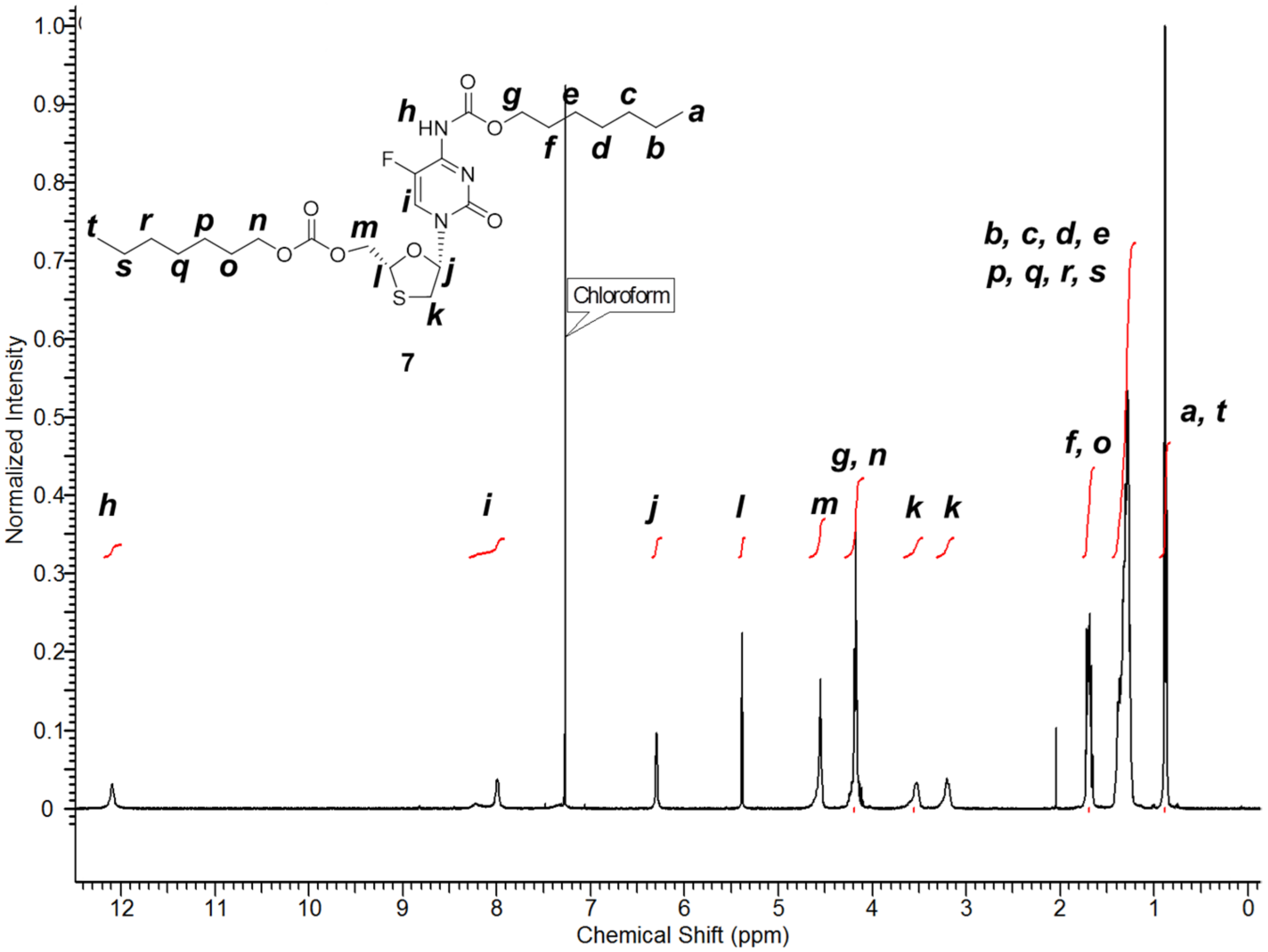

Supplementary Figure 7. ^{1}H NMR (500 MHz) of **7** in $CDCl_3$.

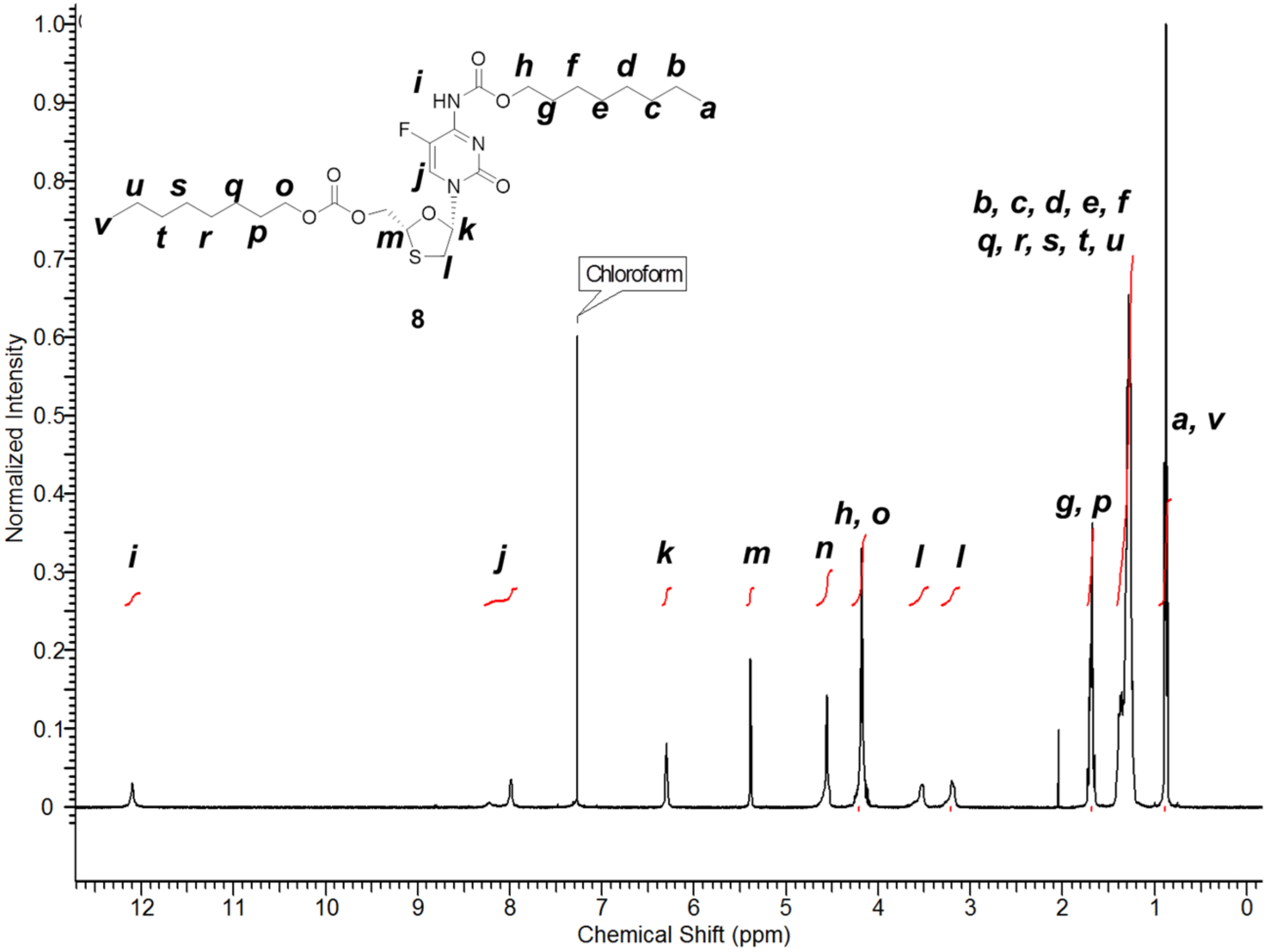

Supplementary Figure 8. ^{1}H NMR (500 MHz) of **8** in $CDCl_3$.

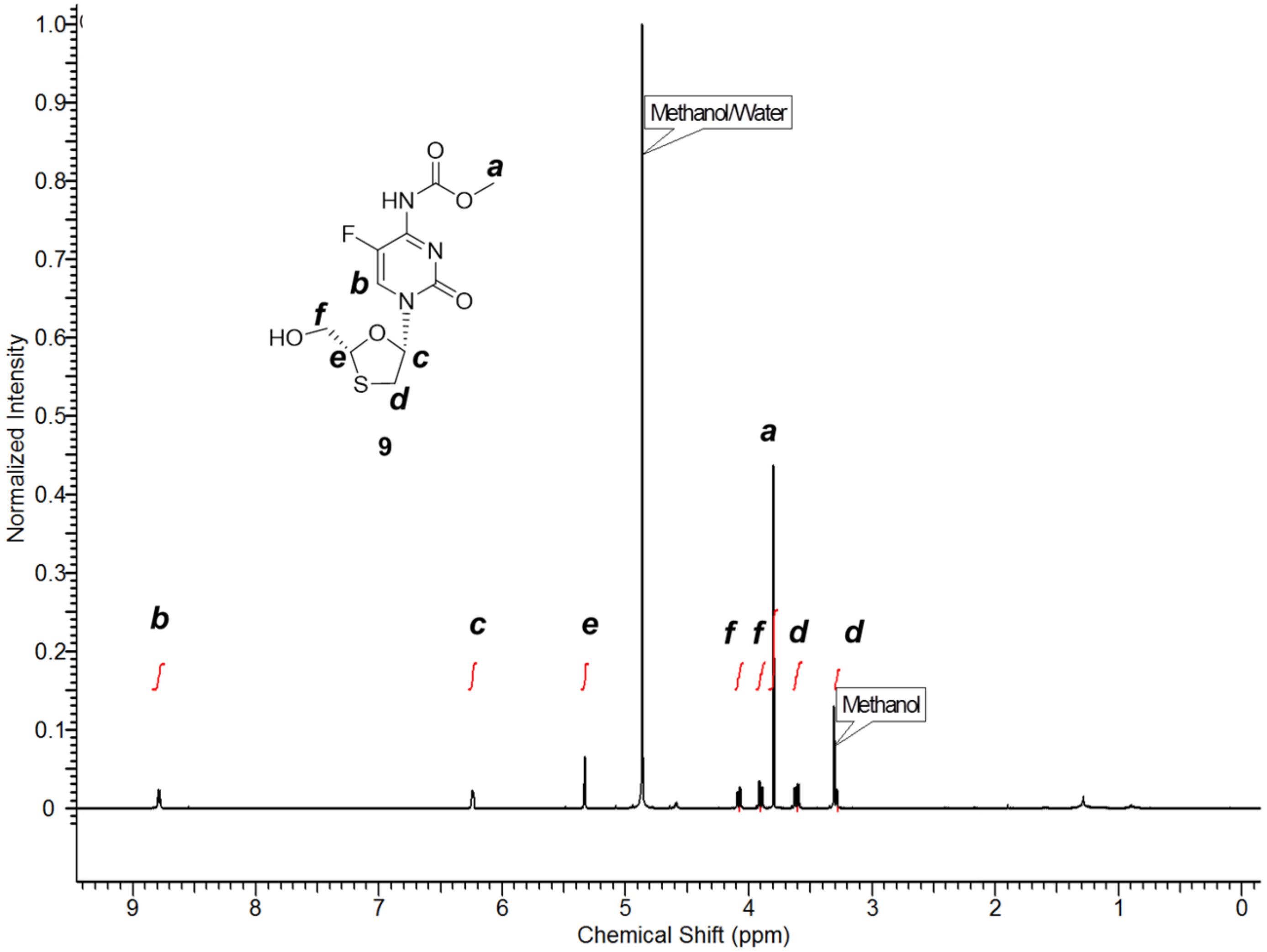

Supplementary Figure 9. ^{1}H NMR (500 MHz) of **9** in CD_3OD.

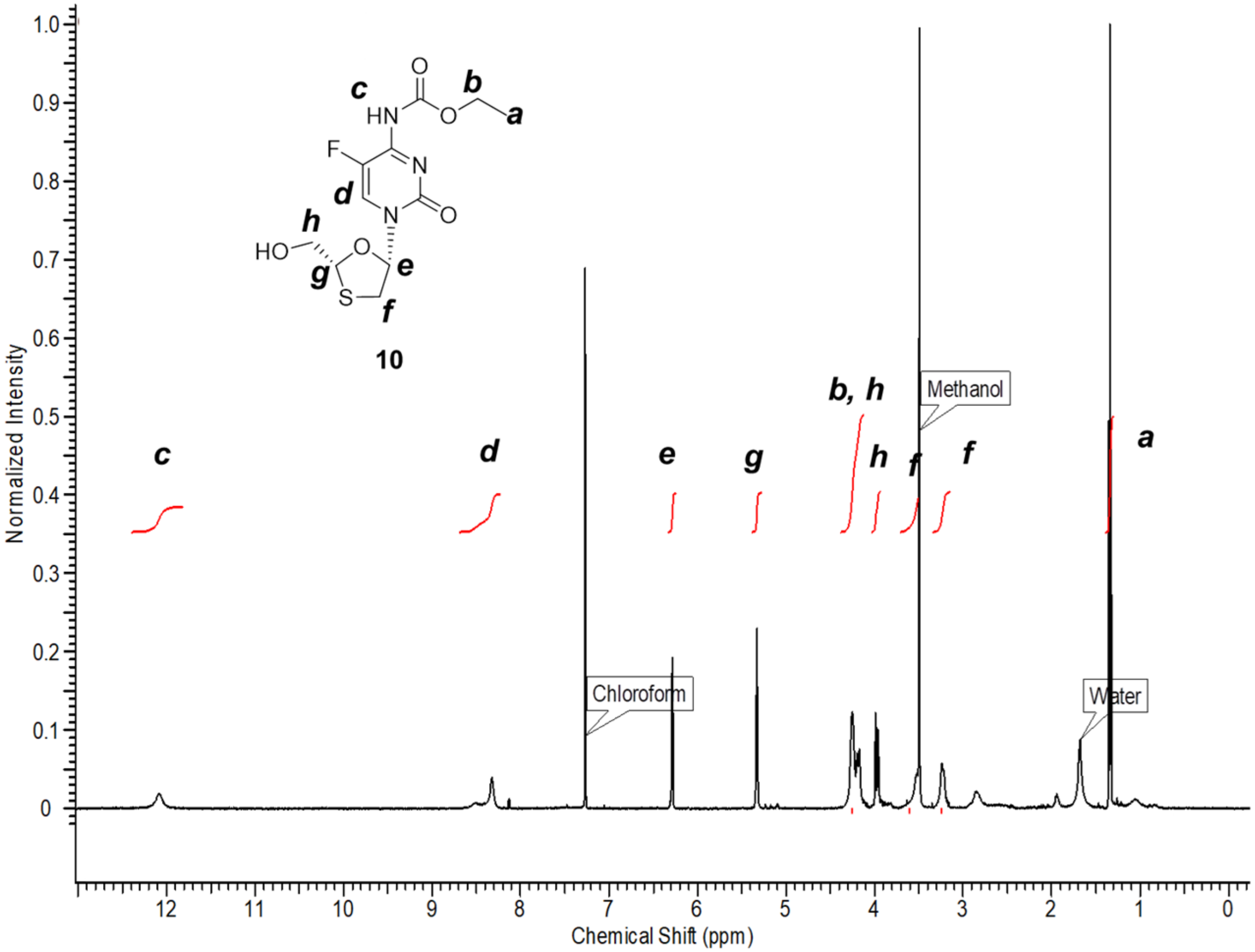

Supplementary Figure 10. ^{1}H NMR (500 MHz) of **10** in $CDCl_3$.

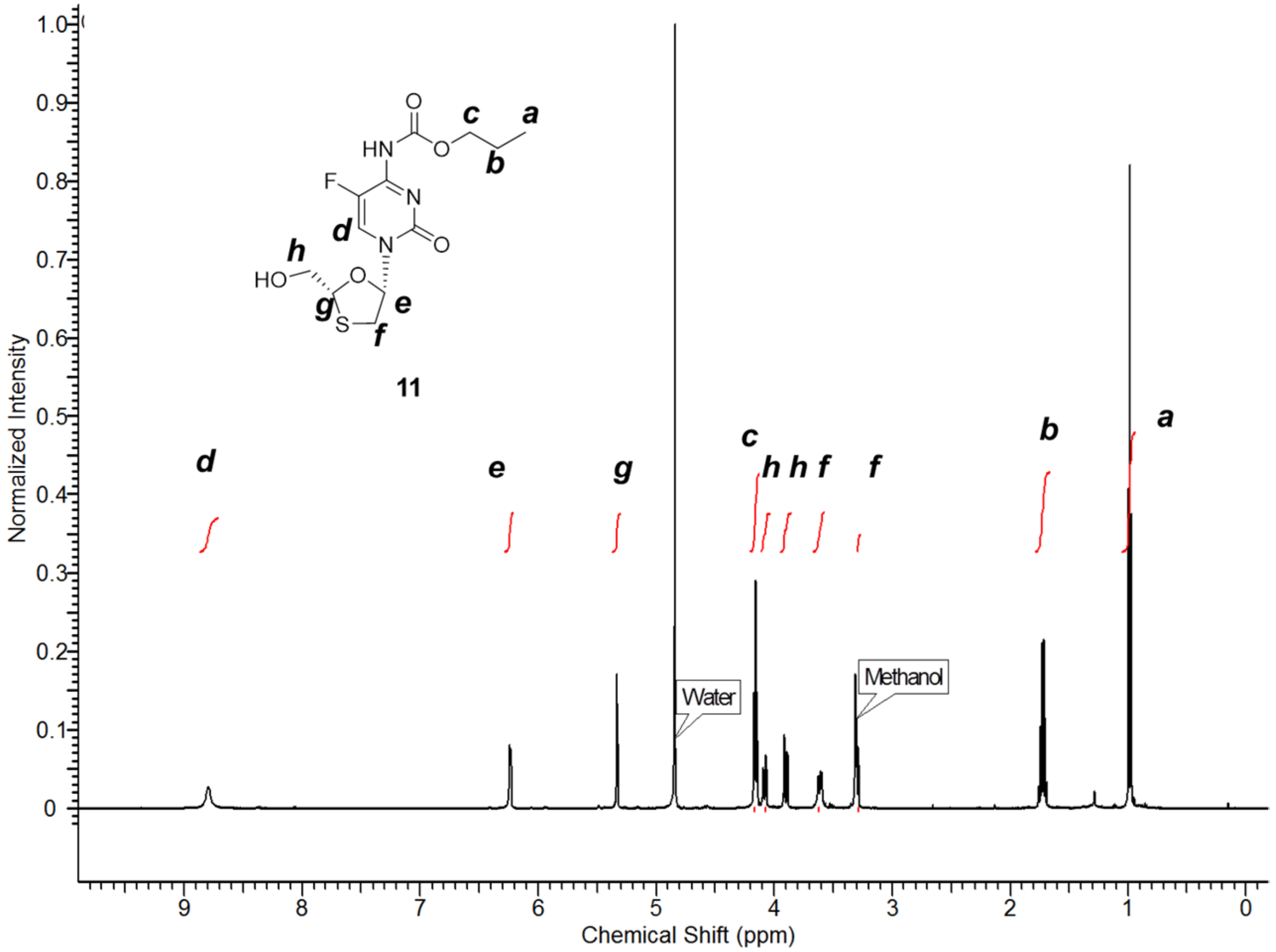

Supplementary Figure 11. ^{1}H NMR (500 MHz) of **11** in CD_3OD.

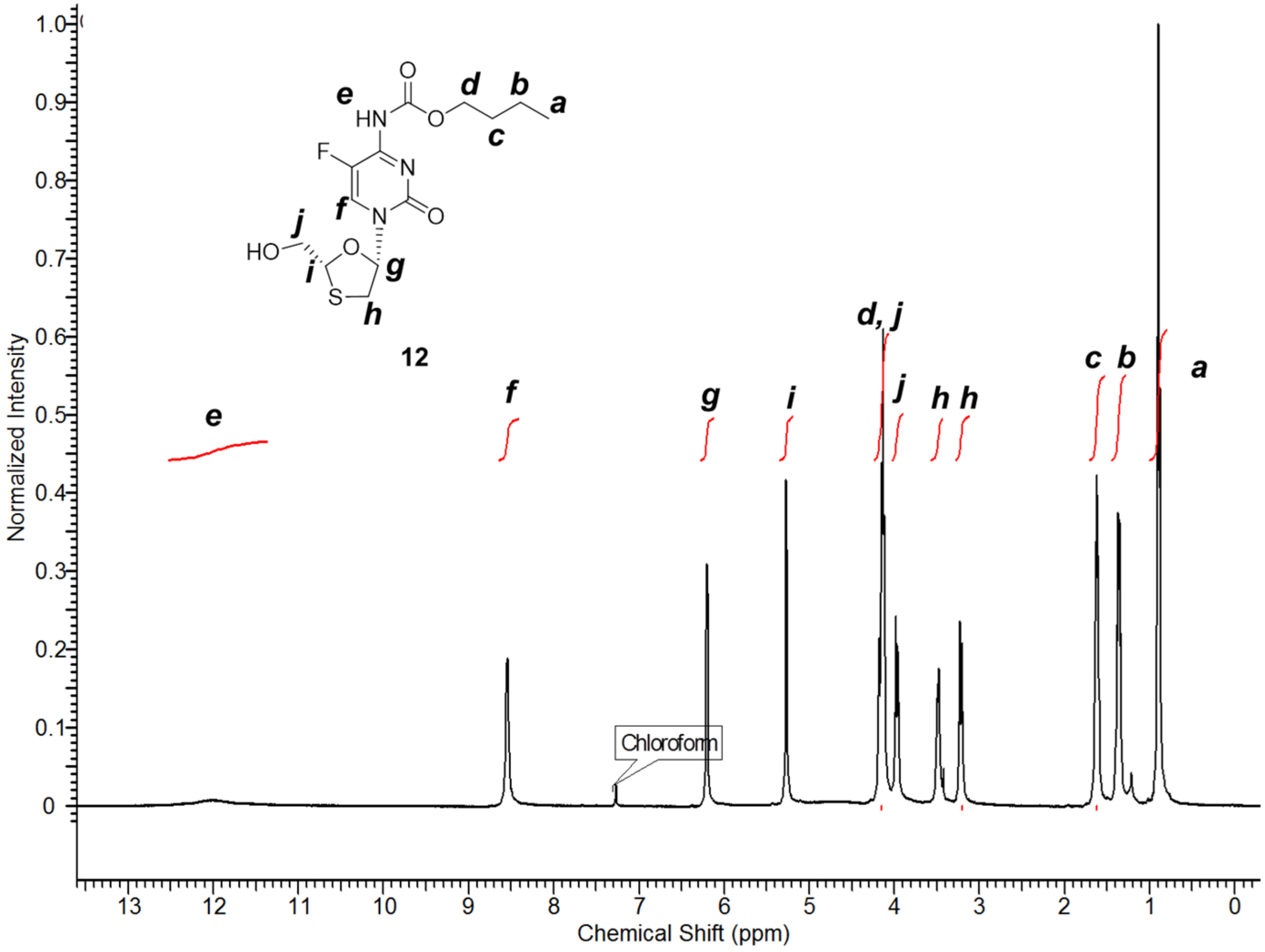

Supplementary Figure 12. ^{1}H NMR (500 MHz) of **12** in $CDCl_3$.

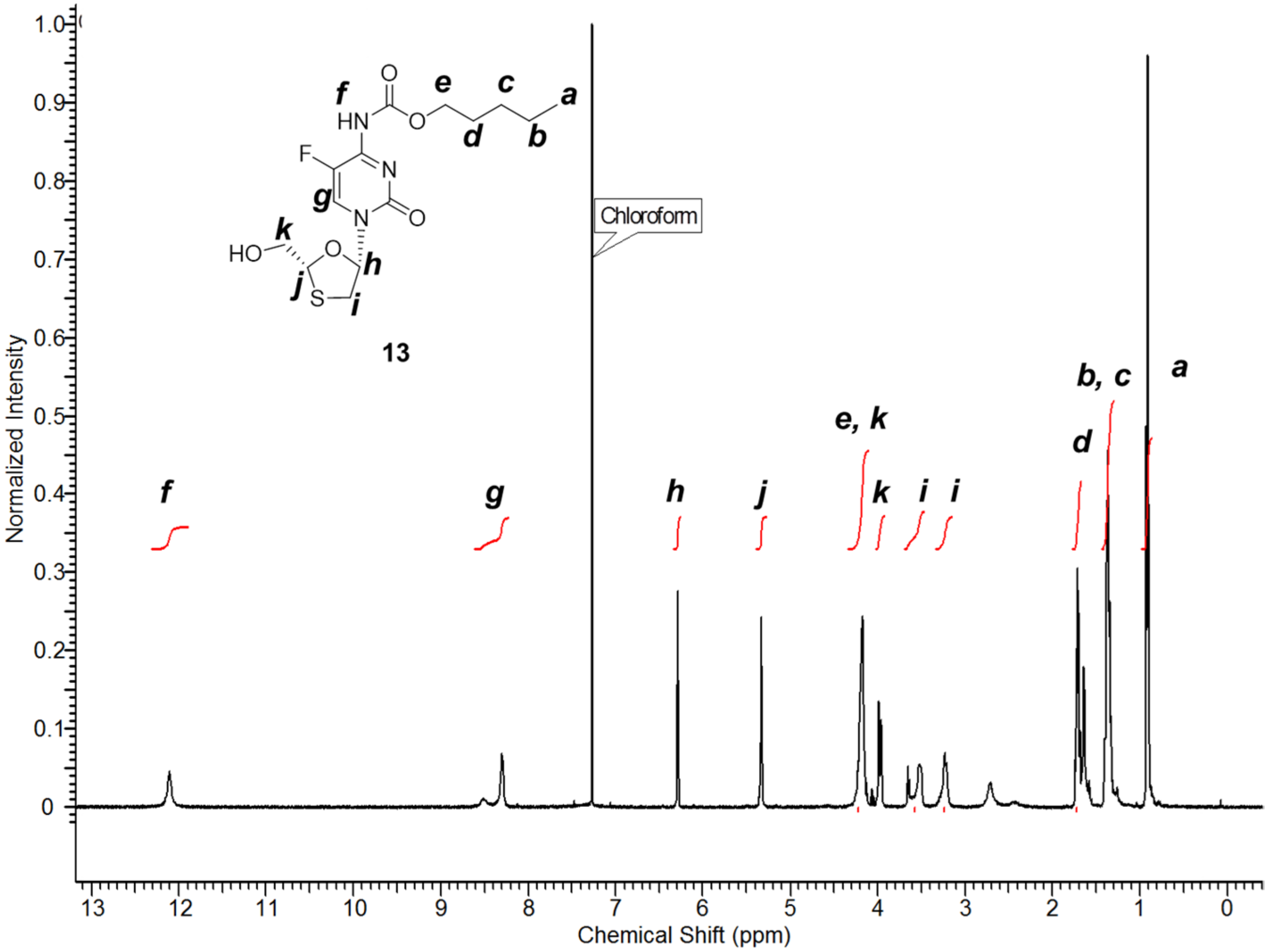

Supplementary Figure 13. ^{1}H NMR (500 MHz) of **13** in $CDCl_3$.

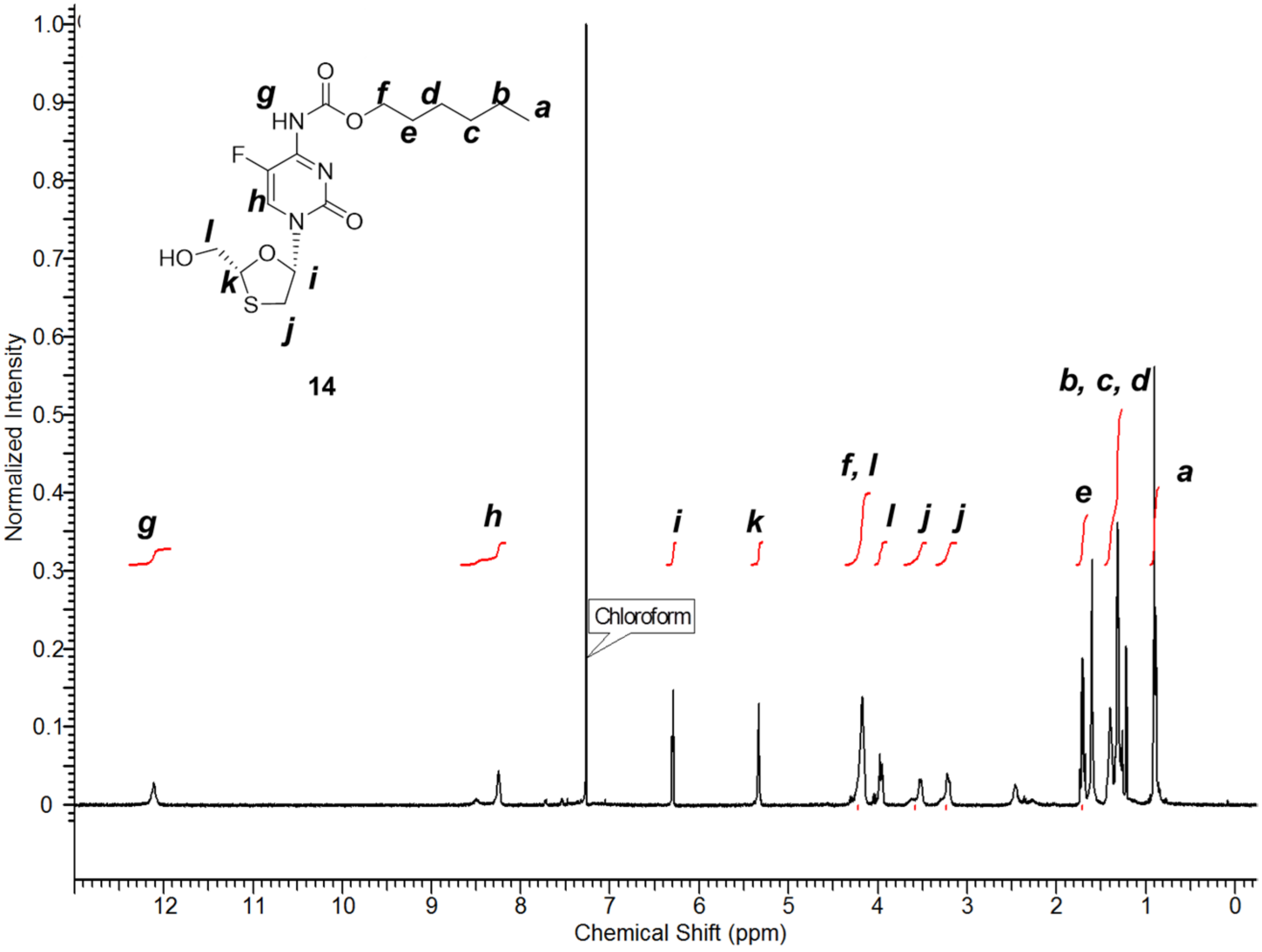

Supplementary Figure 14. ^{1}H NMR (500 MHz) of **14** in $CDCl_3$.

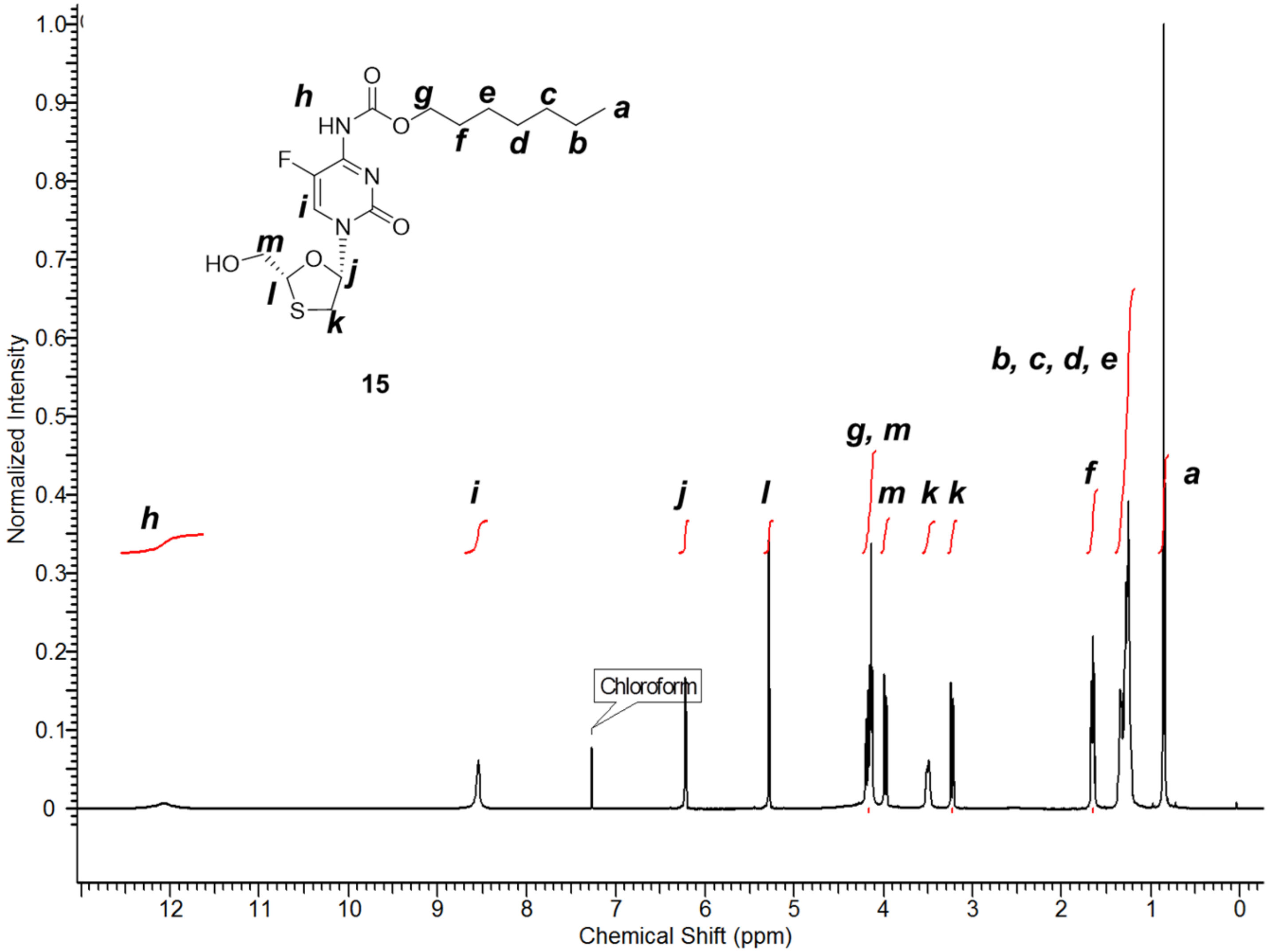

Supplementary Figure 15. ^{1}H NMR (500 MHz) of **15** in $CDCl_3$.

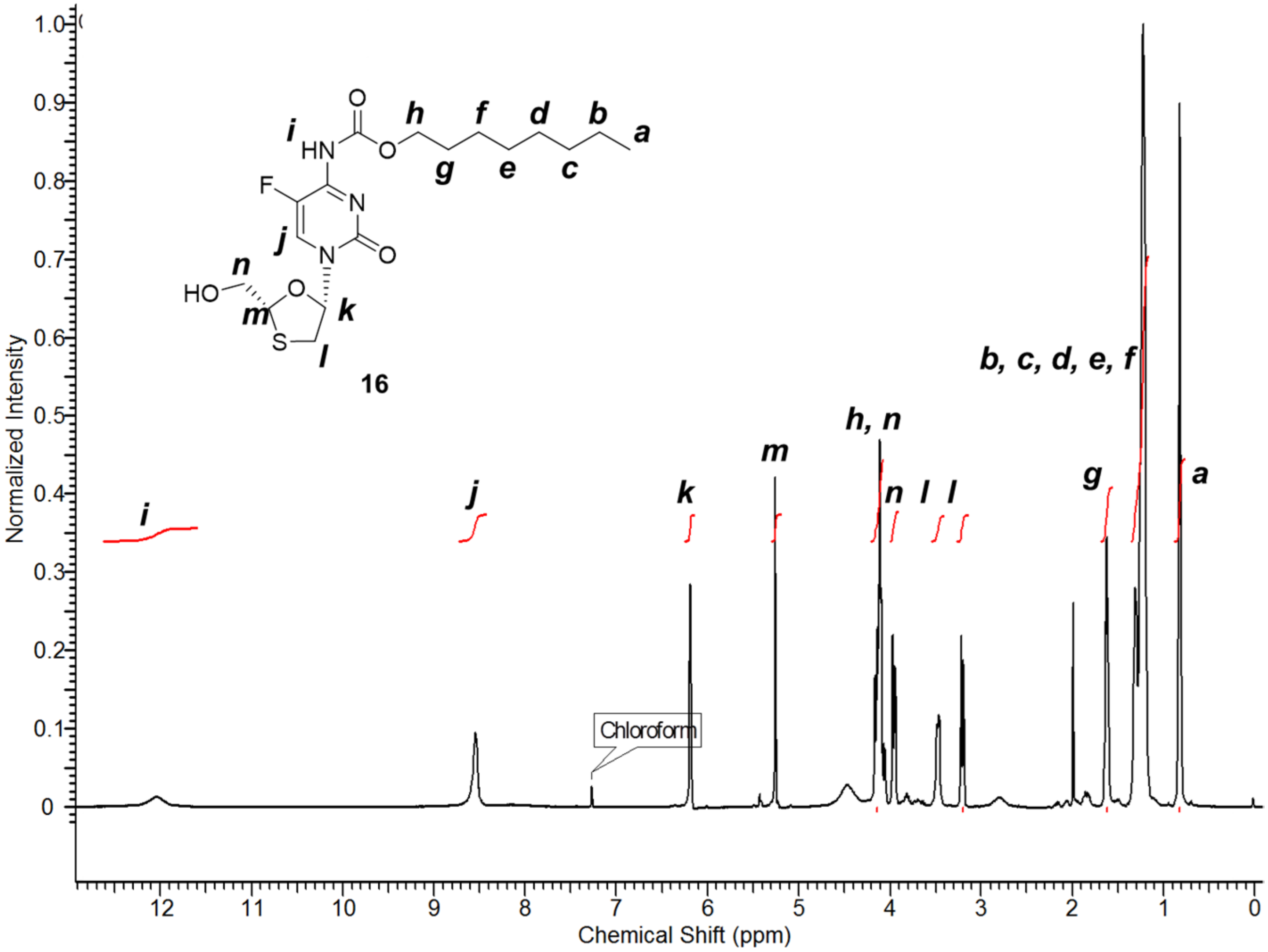

Supplementary Figure 16. ^{1}H NMR (500 MHz) of **16** in $CDCl_3$

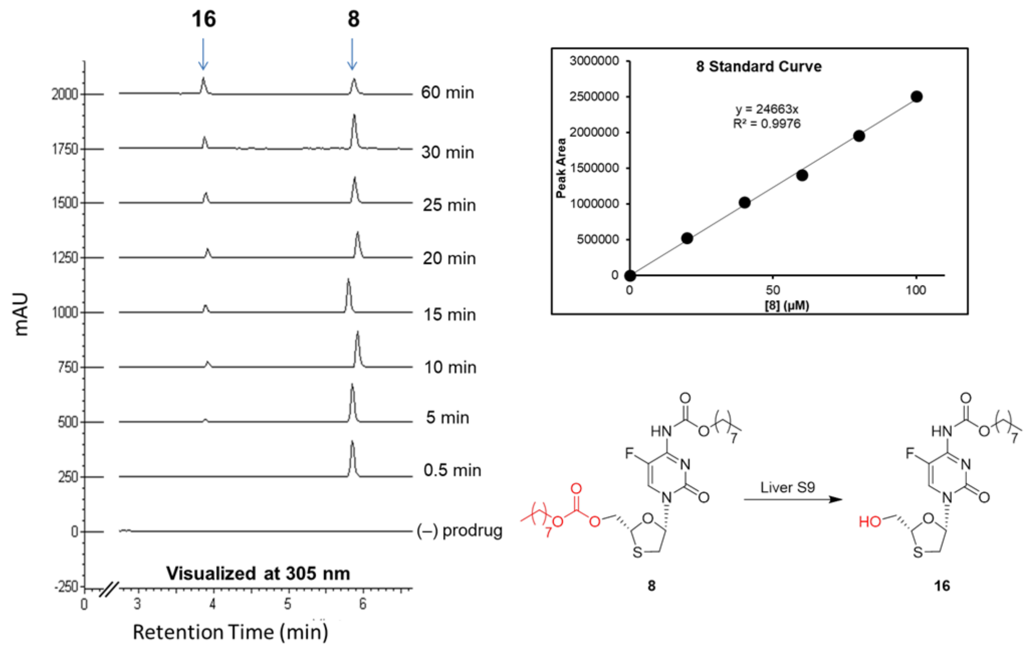

Supplementary Figure 17. Representative HPLC stackplot and standard curve at 305 nm (λ_{max} of FTC carbamates) showing cleavage of **8** to **16** in liver S9 fractions.

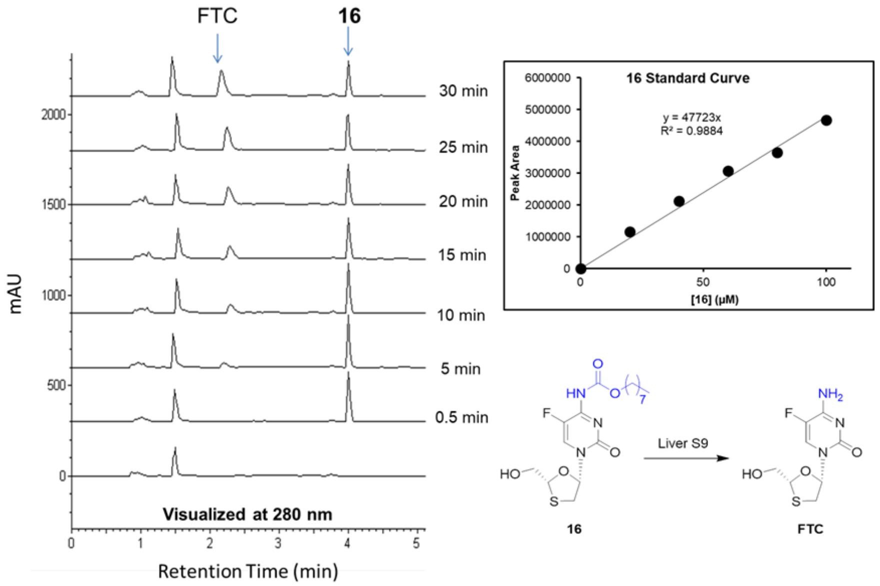

Supplementary Figure 18. Representative HPLC stackplot at 280 nm (λ_{max} of FTC) and standard curve at 305 nm (λ_{max} of FTC carbamates) showing cleavage of **16** to FTC in liver S9 fractions.

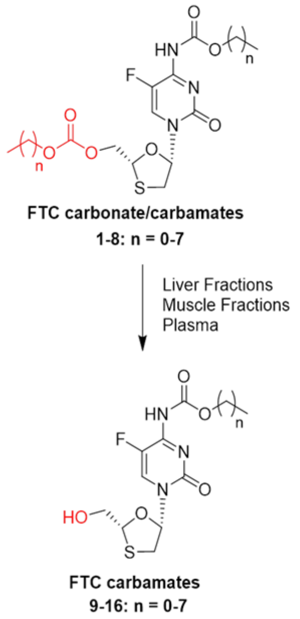

Compound	Liver S9 initial rate (nmol/h/mg)	Muscle S9 initial rate (nmol/h/mg)	Plasma $t_{1/2}$ (h)
1	466 ± 18	7.0 ± 0.2	5.7 ± 0.1
2	847 ± 23	26.4 ± 0.8	0.73 ± 0.02
3	2584 ± 186	114 ± 14	0.63 ± 0.01
4	1293 ± 30	64.9 ± 1.2	2.0 ± 0.0
5	991 ± 31	43.6 ± 3.2	4.2 ± 0.1
6	530 ± 21	26.5 ± 1.6	5.2 ± 0.2
7	191 ± 13	17.7 ± 1.6	7.1 ± 0.3
8	124 ± 4	11.9 ± 0.2	12.1 ± 0.5

Supplementary Figure 19. Calculated initial rates (at 1 mM) of carbonate cleavage of **1-8** in human liver and muscle S9 fractions, and half-lives in human plasma.

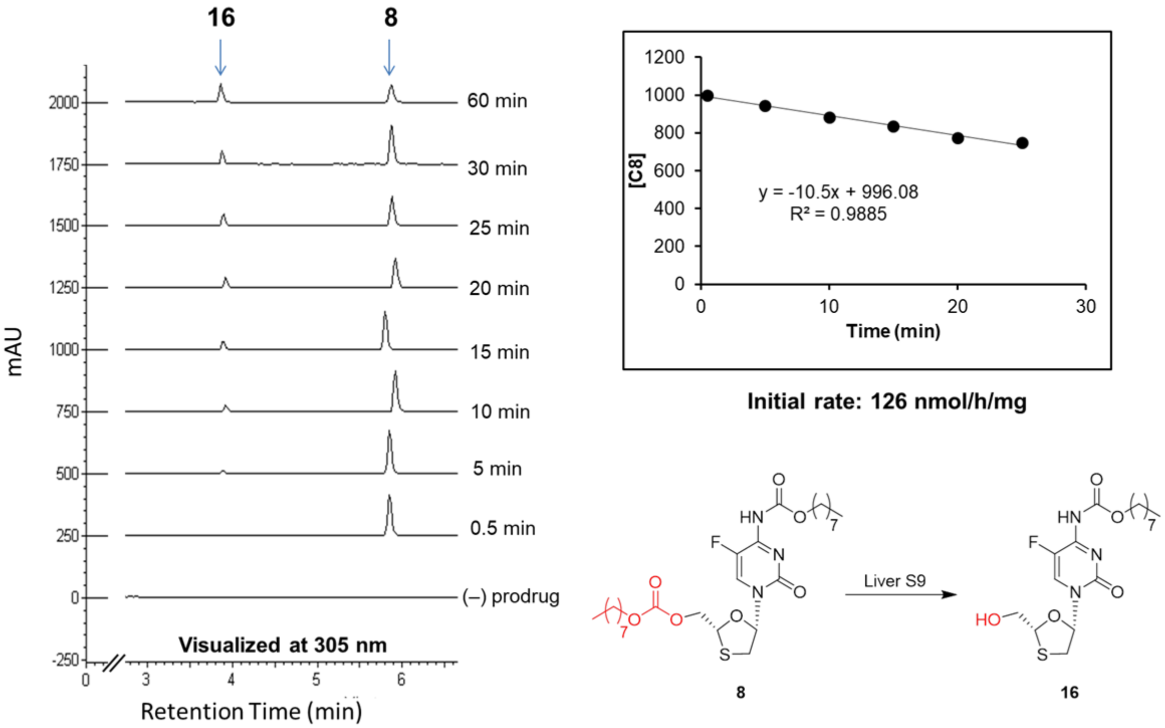

Supplementary Figure 20. Left: Representative HPLC stackplot at 305 nm (λ_{max} of FTC carbamates) and calculated initial rate showing cleavage of **8** to **16** in liver S9 fractions.

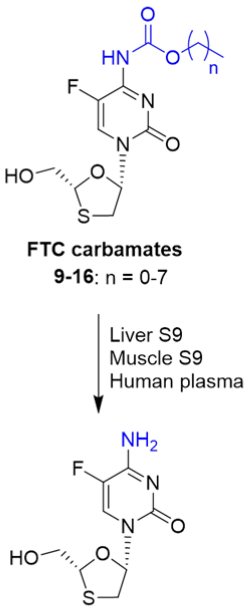

Compound	Liver S9 initial rate (nmol/h/mg)	Muscle S9 initial rate (nmol/h/mg)	Plasma $t_{1/2}$ (h)
**9**	1.5 ± 0.6	0.16 ± 0.01	53 ± 4
**10**	4.8 ± 1.5	0.10 ± 0.02	48 ± 14
**11**	22 ± 1	0.38 ± 0.05	41 ± 2
**12**	36 ± 7	0.38 ± 0.08	23 ± 1
Capecitabine	36 ± 4	0.25 ± 0.03	35 ± 5
**13**	73 ± 12	0.51 ± 0.18	14 ± 0
**14**	212 ± 8	0.73 ± 0.10	15 ± 1
**15**	123 ± 13	0.91 ± 0.06	17 ± 2
**16**	70 ± 7	0.87 ± 0.01	16 ± 1

Supplementary Figure 21. Calculated initial rates (at 1 mM) of carbamate cleavage of **9-16** and capecitabine in human liver and muscle S9 fractions, and half-lives in human plasma.

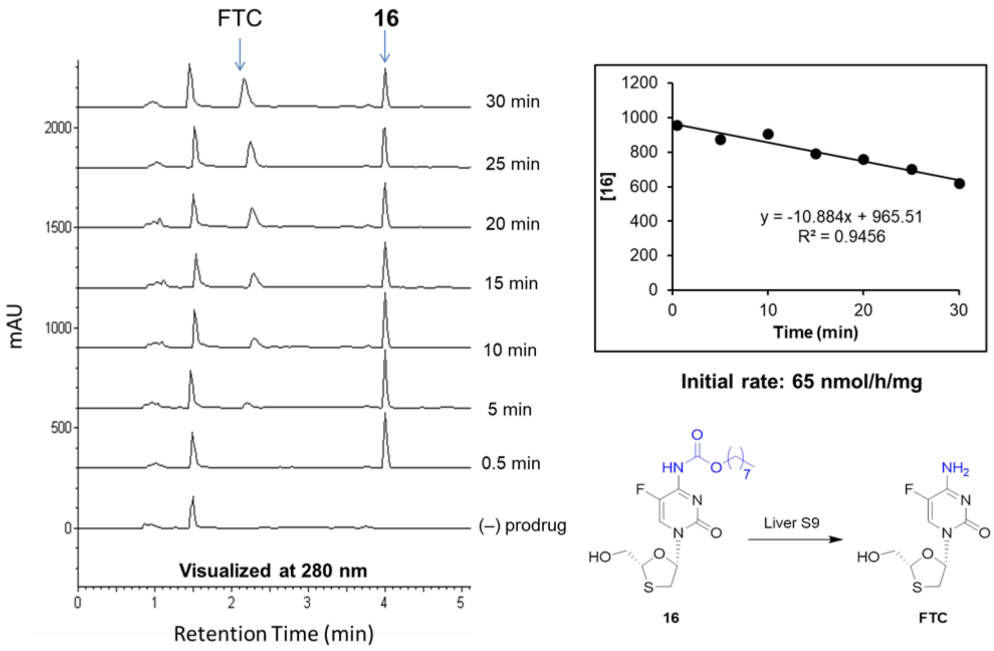

Supplementary Figure 22. Left: Representative HPLC stackplot at 280 nm (λ_{max} of FTC) and calculated initial rate showing cleavage of **16** to FTC in human liver S9 fractions.

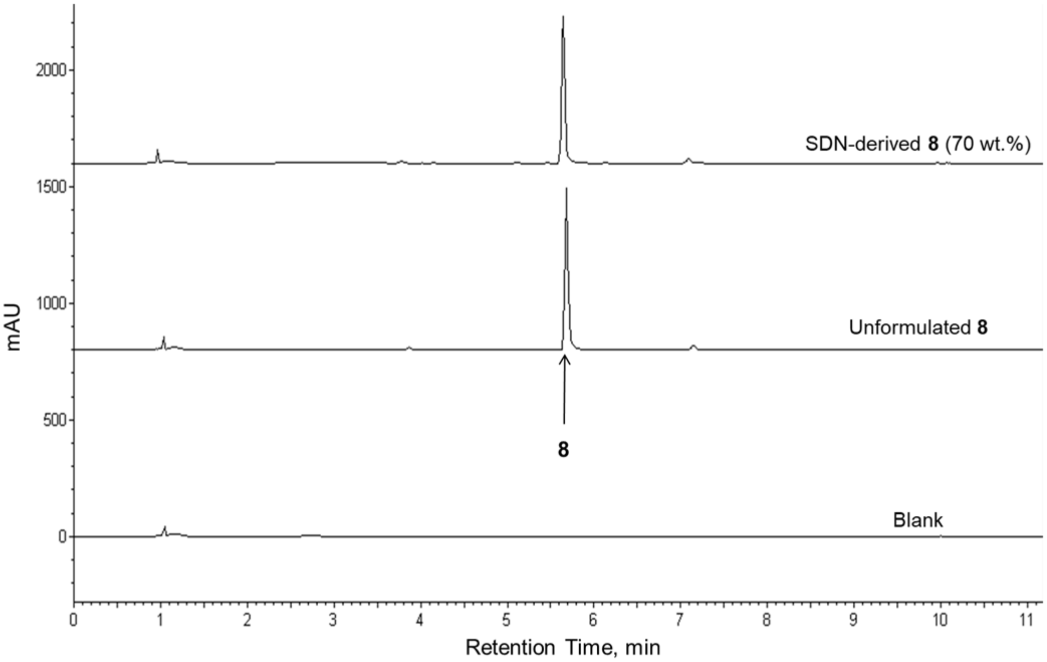

Supplementary Figure 23. Representative HPLC stackplot at 305 nm (λ_{max} of FTC carbamates) showing **8** derived from SSPNs relative to an unformulated prodrug control at equal concentration.

	F68	PVPk30	HPMC	PEG1K	F127	Kollicoat	PVA	Surfactant "Hits"
TPGS	5	3	4	1	4	2	3	22
Tween 20	4	8	6	5	4	5	6	38
Tween 80	4	4	4	4	7	3	6	32
NDC	6	8	9	7	5	7	6	48
AOT	6	5	5	6	6	4	5	37
Solutol	4	6	5	4	7	2	5	33
Polymer "Hits"	29	34	33	27	33	23	31	

Supplementary Figure 24. The number of hits for each binary combination of polymer and surfactant that occurred across the combined SSPN libraries at 10 wt% loading of prodrug. The central cells indicate the number of times an exact combination appeared as a SSPN hit, while the bottom and furthest right column show the total number of SSPN hits in which a given single excipient appears.

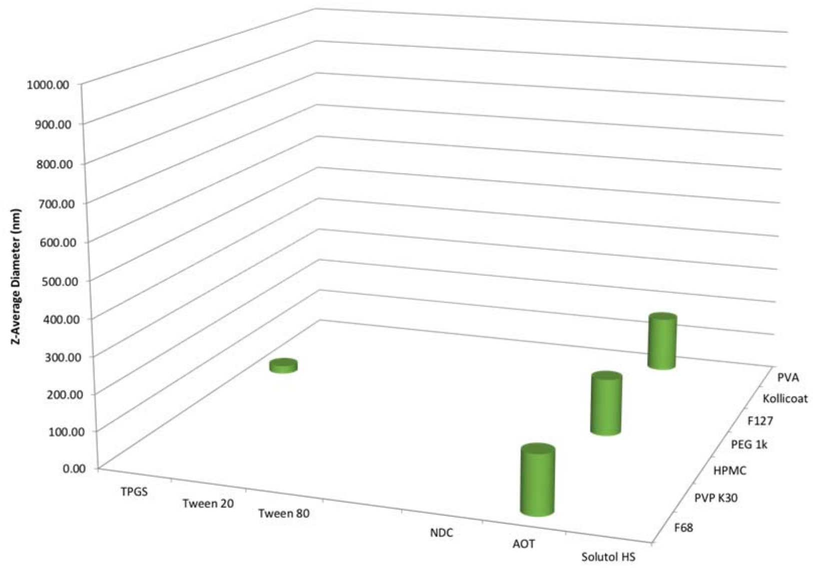

Supplementary Figure 25. Number of SSPN "hits" generated from a library of 42 binary combinations of polymer and surfactant, with prodrug **1** at 10 wt% loading relative to excipients. Data is shown as the average of 3 DLS scans for the z-average hydrodynamic diameter, with only those determined as 'hits' being shown. A 'hit' was confirmed if re-dispersion was easily achieved at 1 mg/mL, z-average diameter <1000nm, and polydispersity index <0.4

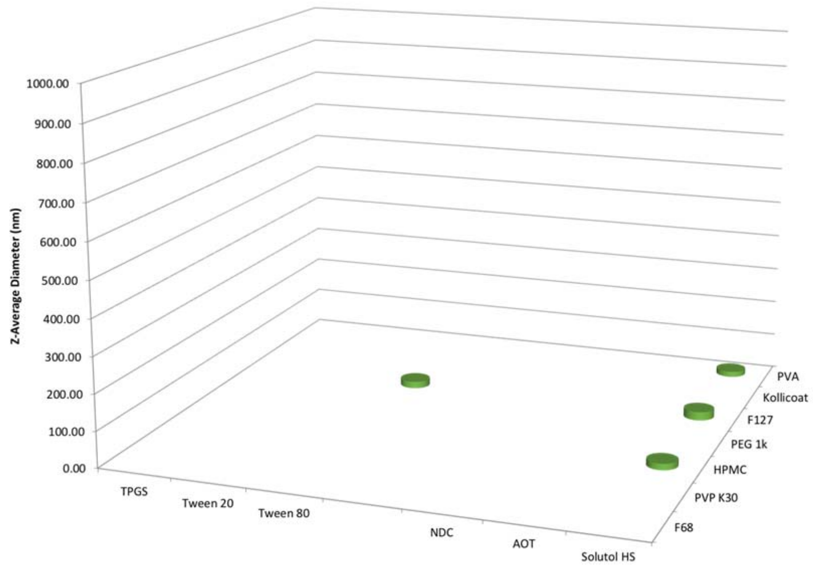

Supplementary Figure 26. Number of SSPN "hits" generated from a library of 42 binary combinations of polymer and surfactant, with prodrug **2** at 10 wt% loading relative to excipients. Data is shown as the average of 3 DLS scans for the z-average hydrodynamic diameter, with only those determined as 'hits' being shown. A 'hit' was confirmed if re-dispersion was easily achieved at 1 mg/mL, z-average diameter <1000nm, and polydispersity index <0.4

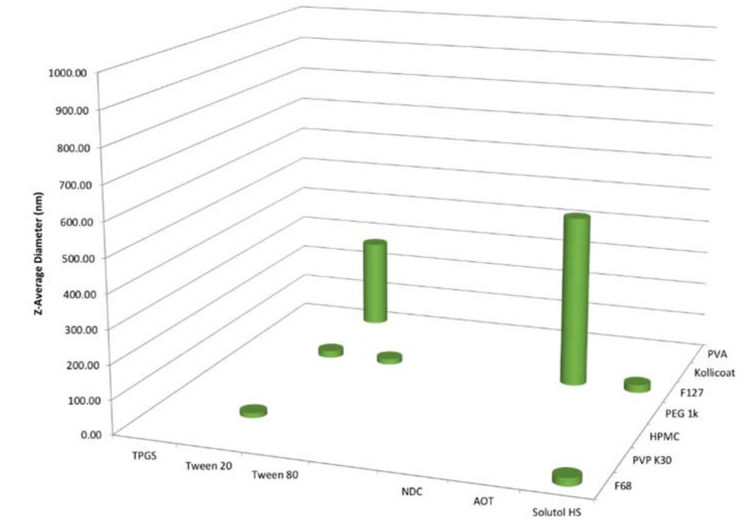

Supplementary Figure 27. Number of SSPN "hits" generated from a library of 42 binary combinations of polymer and surfactant, with prodrug **3** at 10 wt% loading relative to excipients. Data is shown as the average of 3 DLS scans for the z-average hydrodynamic diameter, with only those determined as 'hits' being shown. A 'hit' was confirmed if re-dispersion was easily achieved at 1 mg/mL, z-average diameter <1000nm, and polydispersity index <0.4

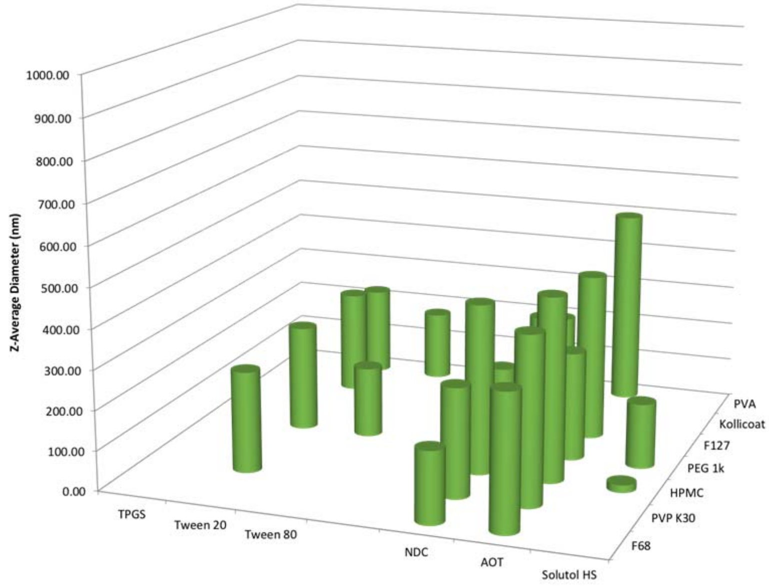

Supplementary Figure 28. Number of SSPN "hits" generated from a library of 42 binary combinations of polymer and surfactant, with prodrug **4** at 10 wt% loading relative to excipients. Data is shown as the average of 3 DLS scans for the z-average hydrodynamic diameter, with only those determined as 'hits' being shown. A 'hit' was confirmed if re-dispersion was easily achieved at 1 mg/mL, z-average diameter <1000nm, and polydispersity index <0.4

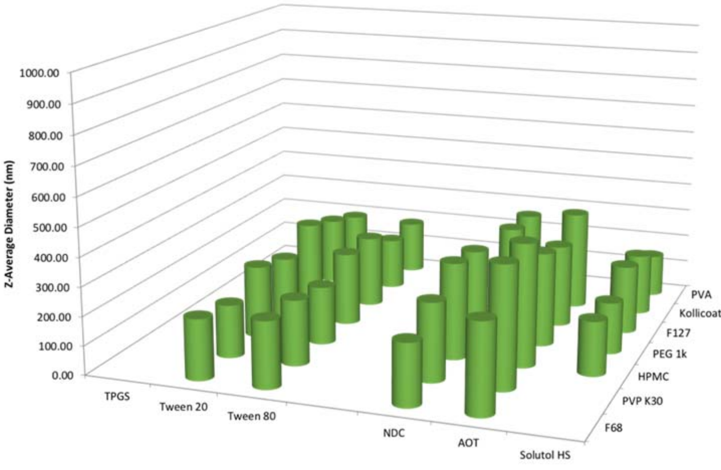

Supplementary Figure 29. Number of SSPN "hits" generated from a library of 42 binary combinations of polymer and surfactant, with prodrug **5** at 10 wt% loading relative to excipients. Data is shown as the average of 3 DLS scans for the z-average hydrodynamic diameter, with only those determined as 'hits' being shown. A 'hit' was confirmed if re-dispersion was easily achieved at 1 mg/mL, z-average diameter <1000nm, and polydispersity index <0.4

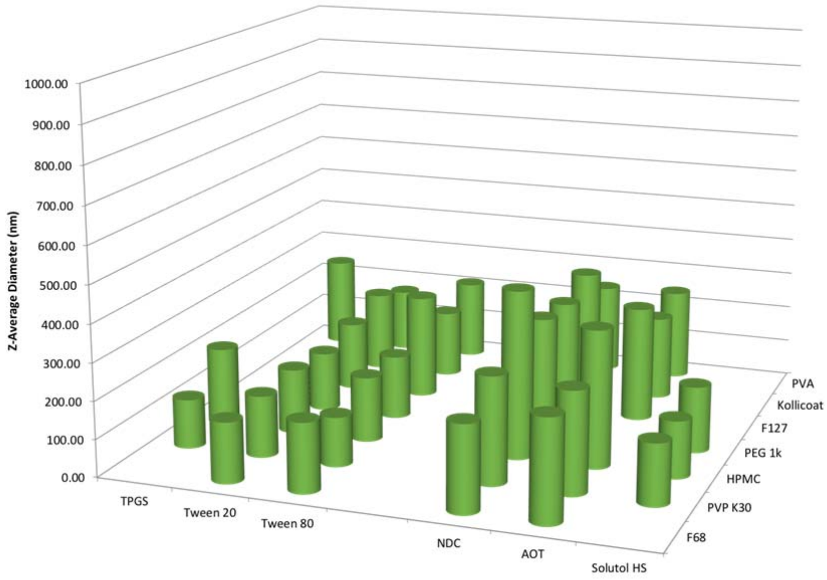

Supplementary Figure 30. Number of SSPN "hits" generated from a library of 42 binary combinations of polymer and surfactant, with prodrug **6** at 10 wt% loading relative to excipients. Data is shown as the average of 3 DLS scans for the z-average hydrodynamic diameter, with only those determined as 'hits' being shown. A 'hit' was confirmed if re-dispersion was easily achieved at 1 mg/mL, z-average diameter <1000nm, and polydispersity index <0.4

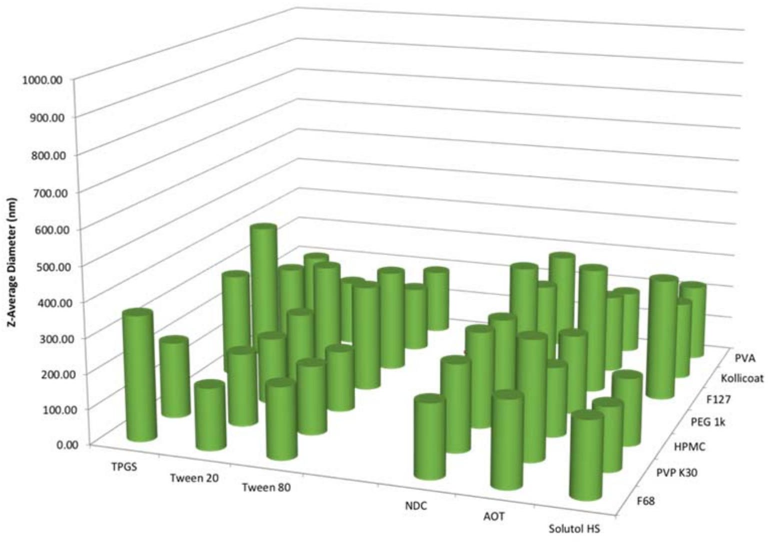

Supplementary Figure 31. Number of SSPN "hits" generated from a library of 42 binary combinations of polymer and surfactant, with prodrug **7** at 10 wt% loading relative to excipients. Data is shown as the average of 3 DLS scans for the z-average hydrodynamic diameter, with only those determined as 'hits' being shown. A 'hit' was confirmed if re-dispersion was easily achieved at 1 mg/mL, z-average diameter <1000nm, and polydispersity index <0.4

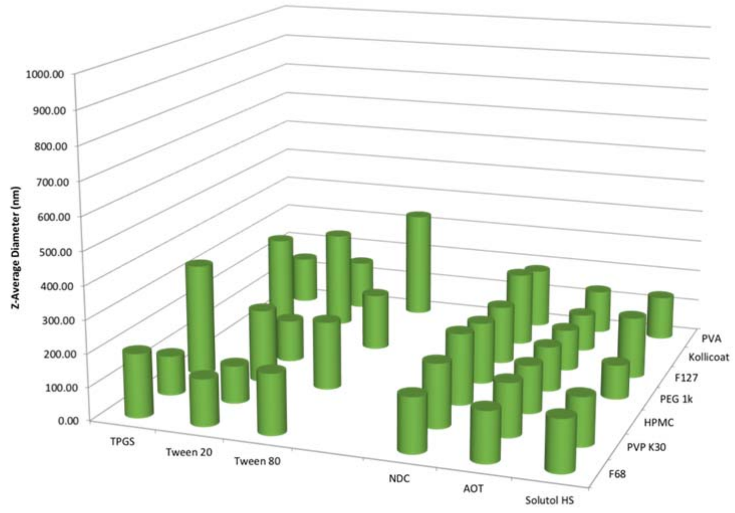

Supplementary Figure 32. Number of SSPN "hits" generated from a library of 42 binary combinations of polymer and surfactant, with prodrug **8** at 10 wt% loading relative to excipients. Data is shown as the average of 3 DLS scans for the z-average hydrodynamic diameter, with only those determined as 'hits' being shown. A 'hit' was confirmed if re-dispersion was easily achieved at 1 mg/mL, z-average diameter <1000nm, and polydispersity index <0.4

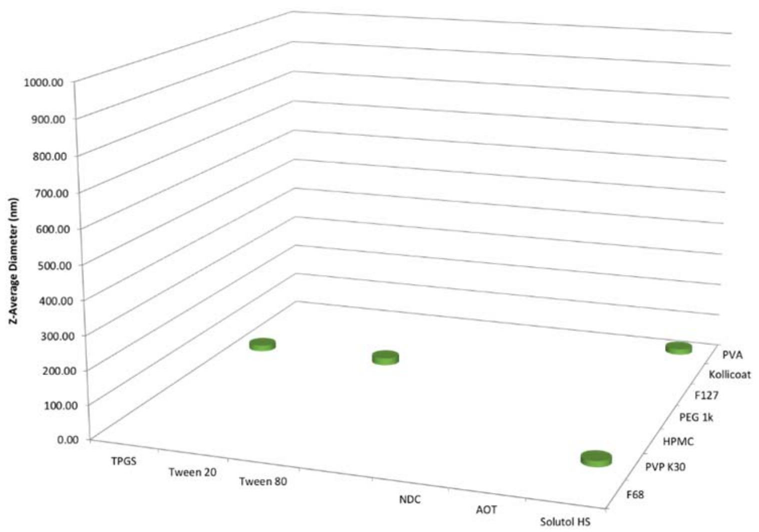

Supplementary Figure 33. Number of SSPN "hits" generated from a library of 42 binary combinations of polymer and surfactant, with prodrug **10** at 10 wt% loading relative to excipients. Data is shown as the average of 3 DLS scans for the z-average hydrodynamic diameter, with only those determined as 'hits' being shown. A 'hit' was confirmed if re-dispersion was easily achieved at 1 mg/mL, z-average diameter <1000nm, and polydispersity index <0.4

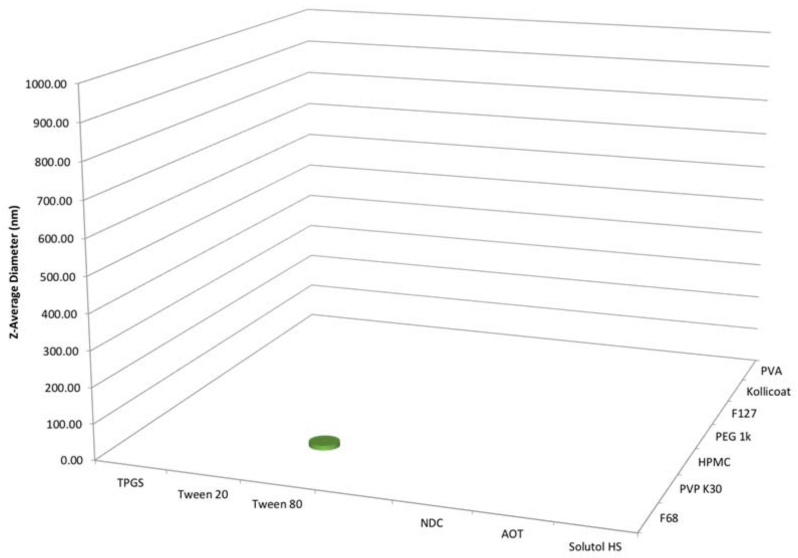

Supplementary Figure 34. Number of SSPN "hits" generated from a library of 42 binary combinations of polymer and surfactant, with prodrug **11** at 10 wt% loading relative to excipients. Data is shown as the average of 3 DLS scans for the z-average hydrodynamic diameter, with only those determined as 'hits' being shown. A 'hit' was confirmed if re-dispersion was easily achieved at 1 mg/mL, z-average diameter <1000nm, and polydispersity index <0.4

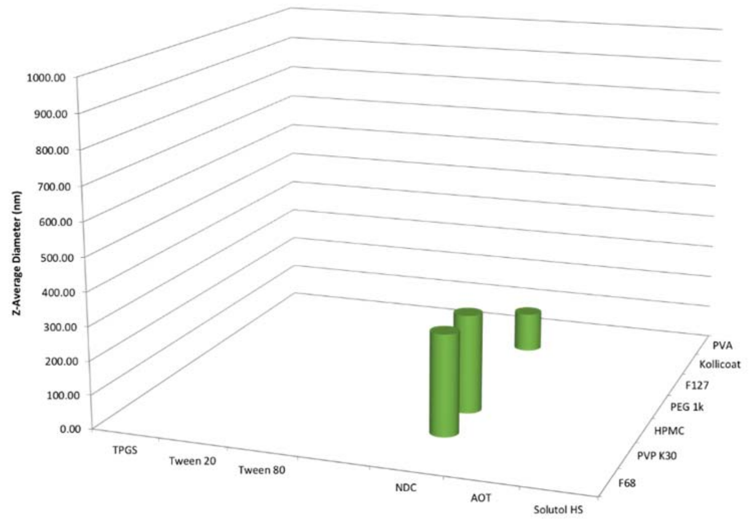

Supplementary Figure 35. Number of SSPN "hits" generated from a library of 42 binary combinations of polymer and surfactant, with prodrug **13** at 10 wt% loading relative to excipients. Data is shown as the average of 3 DLS scans for the z-average hydrodynamic diameter, with only those determined as 'hits' being shown. A 'hit' was confirmed if re-dispersion was easily achieved at 1 mg/mL, z-average diameter <1000nm, and polydispersity index <0.4

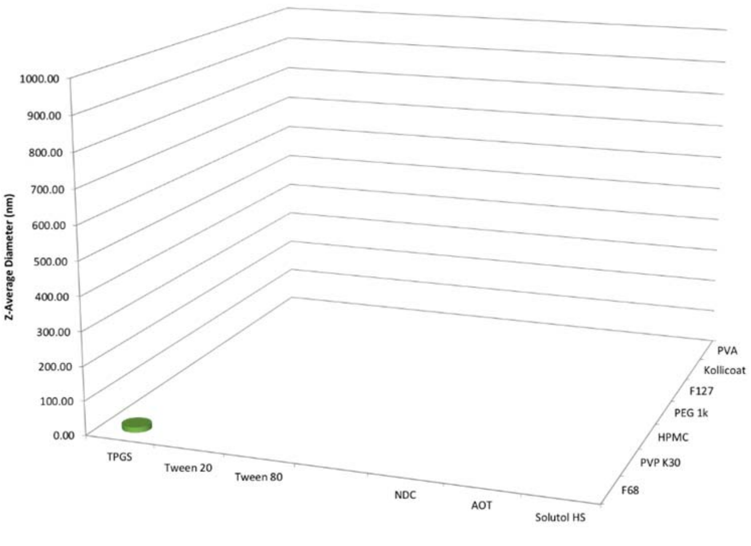

Supplementary Figure 36. Number of SSPN "hits" generated from a library of 42 binary combinations of polymer and surfactant, with prodrug **14** at 10 wt% loading relative to excipients. Data is shown as the average of 3 DLS scans for the z-average hydrodynamic diameter, with only those determined as 'hits' being shown. A 'hit' was confirmed if re-dispersion was easily achieved at 1 mg/mL, z-average diameter <1000nm, and polydispersity index <0.4

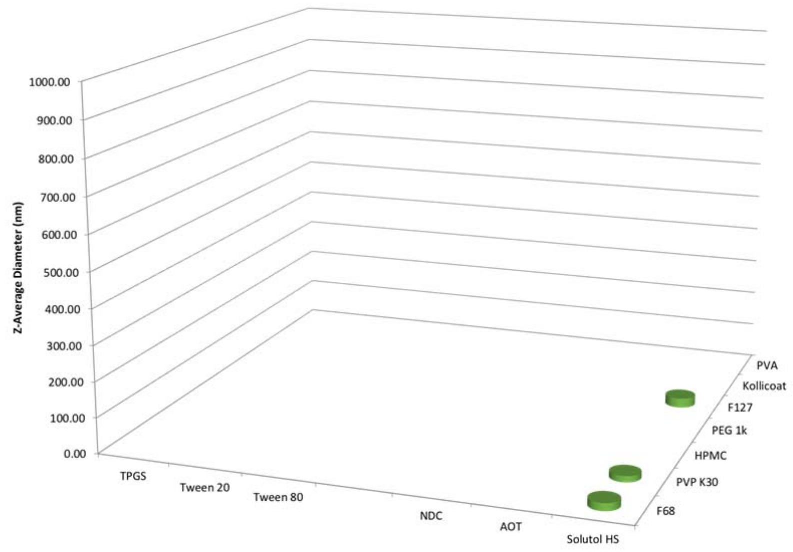

Supplementary Figure 37. Number of SSPN "hits" generated from a library of 42 binary combinations of polymer and surfactant, with prodrug **15** at 10 wt% loading relative to excipients. Data is shown as the average of 3 DLS scans for the z-average hydrodynamic diameter, with only those determined as 'hits' being shown. A 'hit' was confirmed if re-dispersion was easily achieved at 1 mg/mL, z-average diameter <1000nm, and polydispersity index <0.4

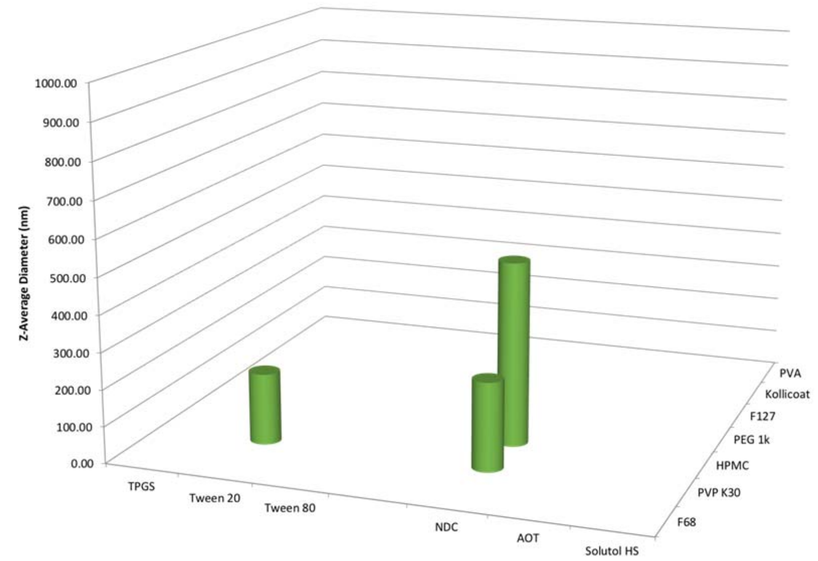

Supplementary Figure 38. Number of SSPN "hits" generated from a library of 42 binary combinations of polymer and surfactant, with prodrug **16** at 10 wt% loading relative to excipients. Data is shown as the average of 3 DLS scans for the z-average hydrodynamic diameter, with only those determined as 'hits' being shown. A 'hit' was confirmed if re-dispersion was easily achieved at 1 mg/mL, z-average diameter <1000nm, and polydispersity index <0.4

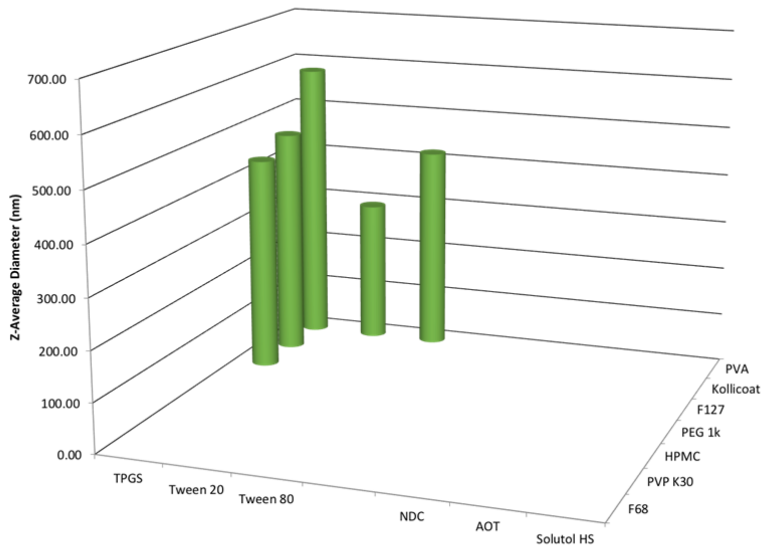

Supplementary Figure 39. Number of SSPN "hits" generated from a library of 42 binary combinations of polymer and surfactant, with prodrug **4** at 50 wt% loading relative to excipients. Data is shown as the average of 3 DLS scans for the z-average hydrodynamic diameter, with only those determined as 'hits' being shown. A 'hit' was confirmed if re-dispersion was easily achieved at 1 mg/mL, z-average diameter <1000nm, and polydispersity index <0.4

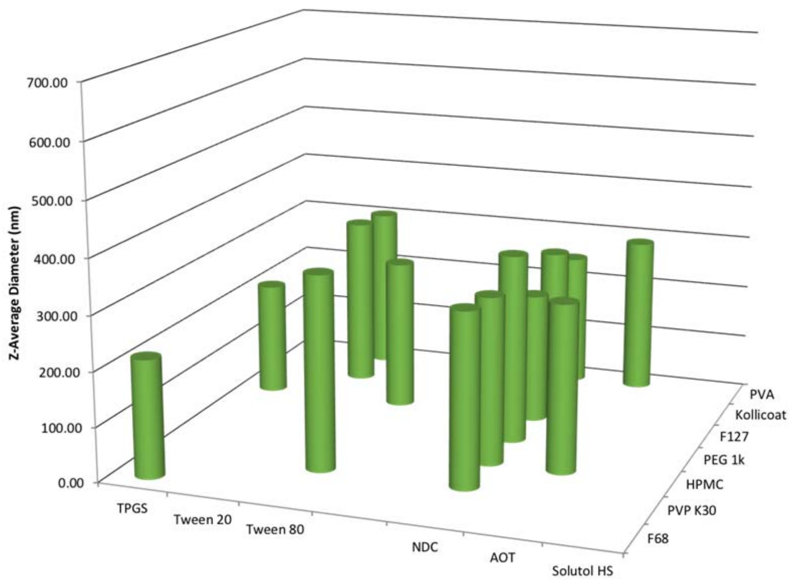

Supplementary Figure 40. Number of SSPN "hits" generated from a library of 42 binary combinations of polymer and surfactant, with prodrug **6** at 50 wt% loading relative to excipients. Data is shown as the average of 3 DLS scans for the z-average hydrodynamic diameter, with only those determined as 'hits' being shown. A 'hit' was confirmed if re-dispersion was easily achieved at 1 mg/mL, z-average diameter <1000nm, and polydispersity index <0.4

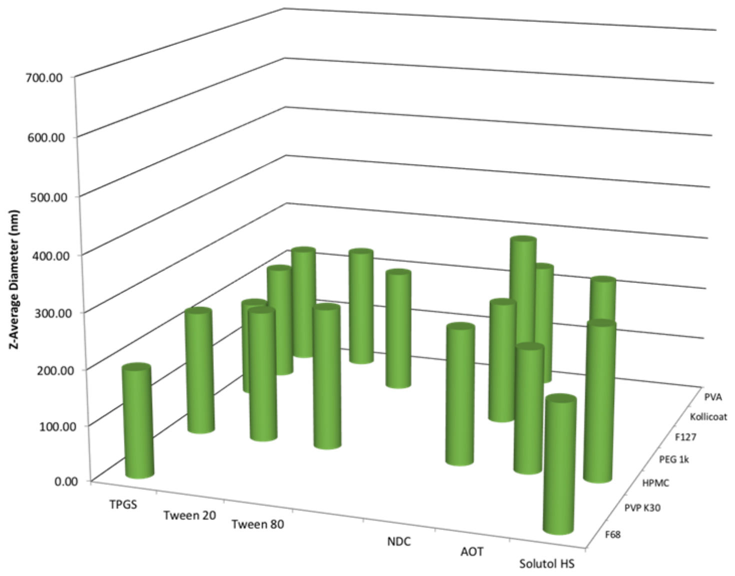

Supplementary Figure 41. Number of SSPN "hits" generated from a library of 42 binary combinations of polymer and surfactant, with prodrug **8** at 50 wt% loading relative to excipients. Data is shown as the average of 3 DLS scans for the z-average hydrodynamic diameter, with only those determined as 'hits' being shown. A 'hit' was confirmed if re-dispersion was easily achieved at 1 mg/mL, z-average diameter <1000nm, and polydispersity index <0.4

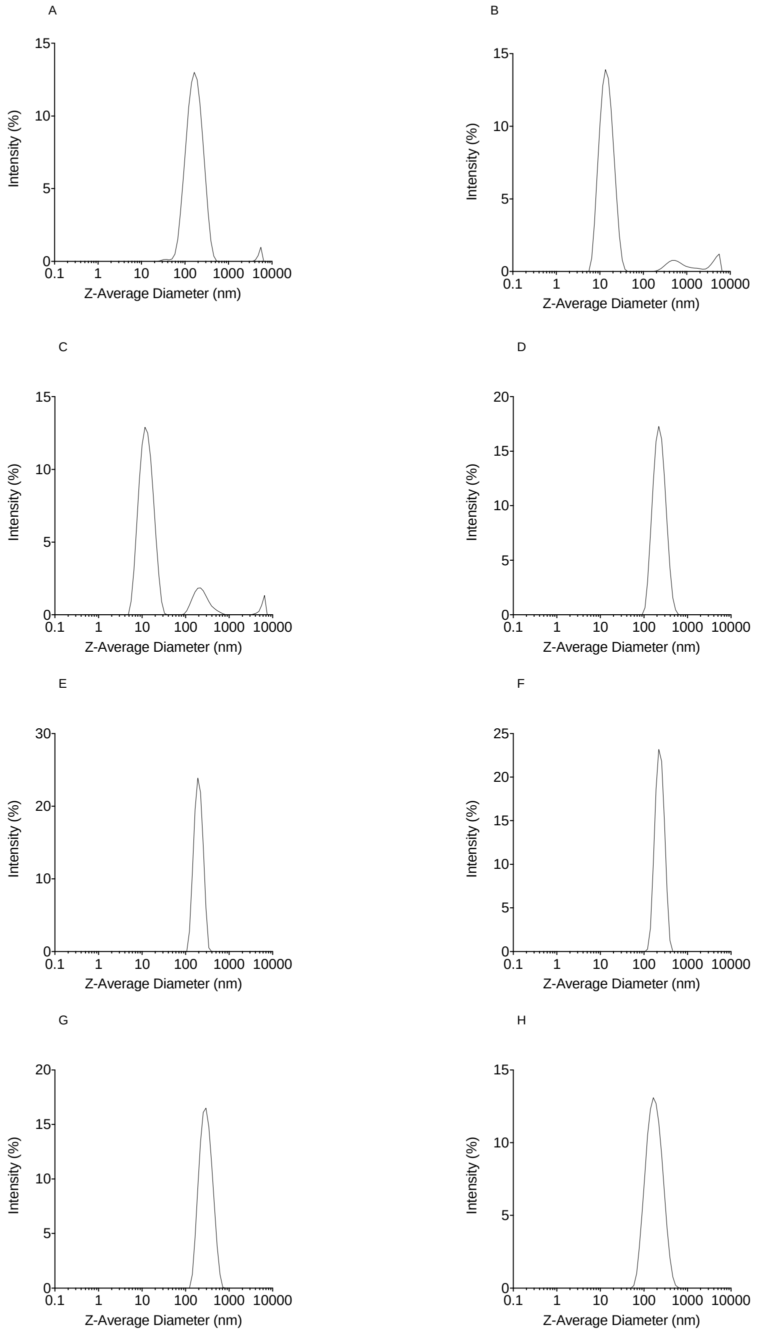

Supplementary Figure 42. Representative Z-Average diameter particle size distribution of selected hits from libraries of 10 wt% loaded SSPNs. Prodrug **1** stabilised by NDC+PVA (a), prodrug **2** stabilised by Solutol + PVA (b), prodrug **3** stabilised by Tween 80 + PVPK30 (c), prodrug **4** stabilised by AOT + PEG1K (d), prodrug **5** stabilised by Tween 20 + PVA (e), prodrug **6** stabilised by NDC + Kollicoat (f), prodrug **7** stabilised by AOT + PVA, and prodrug **8** stabilised by TPGS and PVA. Data is shown as the average of 3 scans from dynamic light scattering analysis.

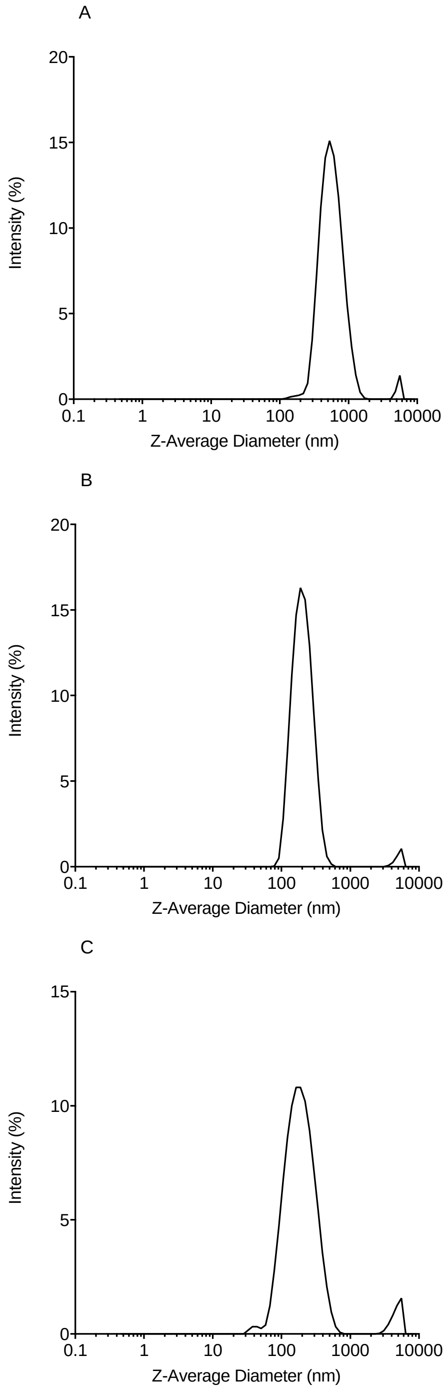

Supplementary Figure 43. Representative Z-Average diameter particle size distribution of selected hits from libraries of 50 wt% loaded SSPNs. Prodrug **4** stabilised by TPGS +PVA (a), prodrug **6** stabilised by TPGS + F127 (b), and prodrug **8** stabilised by TPGS + F127 (c). Data is shown as the average of 3 scans from dynamic light scattering analysis.

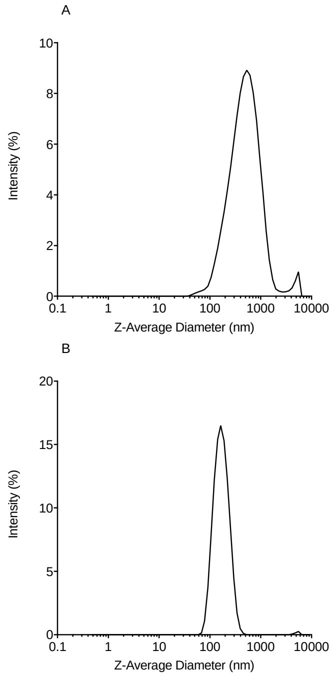

Supplementary Figure 44. Representative Z-Average diameter particle size distribution of selected hits from libraries of 70 wt% loaded SSPNs. Prodrug **4** stabilised by Tween 20 +PVA (a) and prodrug **6** stabilised by AOT + PEG1K (b). Data is shown as the average of 3 scans from dynamic light scattering analysis.

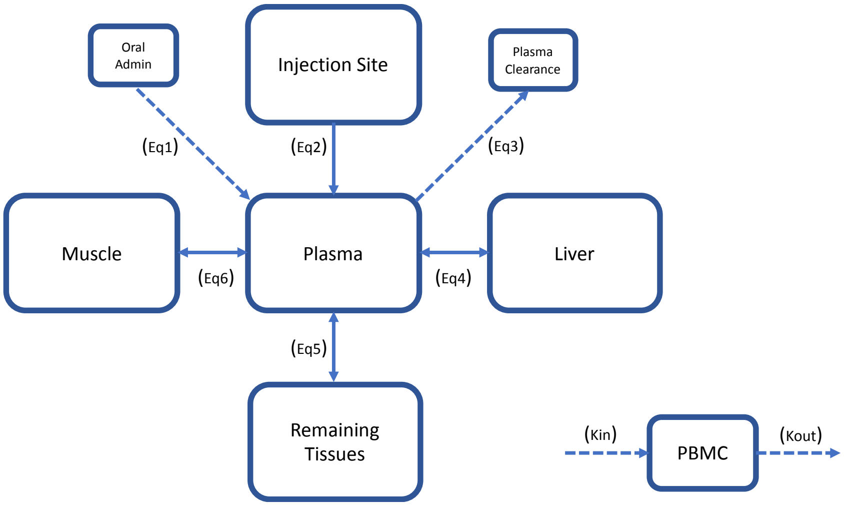

Supplementary Figure 45. Diagram of the IVIVE model describing the systemic distribution of carbonate/carbamate prodrugs, carbamate intermediary and FTC following simulated IM injection. Also shown is the compartments for simulated oral administration and clearance from the plasma. All numbers refer to equations used to describe the flow between compartments. Also shown is the additional module to simulate intracellular FTC-TP concentrations.

Supplementary Table 1. Measured retention times for **1-16**, **FTC**, and **Capecitabine** via the RP-HPLC method described above: 0% to 100% solvent B over 5 minutes at a flow rate of 3 mL min^{-1} (solvent A: Et_3NHOAc (50 mM, pH 8), solvent B: acetonitrile. For prodrugs **1-8** and **9-16**, retention time increases as carbon chain length increases, demonstrating an increase in lipophilicity.

**Compound**	**RP-HPLC Retention Time (min.)**
**FTC**	2.2
**1**	3.0
**2**	3.4
**3**	3.7
**4**	4.2
**5**	4.6
**6**	5.1
**7**	5.4
**8**	5.9
**9**	2.7
**10**	2.9
**11**	3.0
**12**	3.2
**Capecitabine**	3.2
**13**	3.5
**14**	3.5
**15**	3.8
**16**	4.0

Supplementary Table 2. Excipients used to generate libraries of SSPNs consisting of 7 different polymers and 6 different surfactants, resulting in 42 individual binary combinations

**Polymer**	**Surfactant**
Polyethylene glycol 1000 (PEG1K)	Polysorbate 20 (Tween 20)
Polyvinylpyrrolidone K30 (PVPK30)	Polysorbate 80 (Tween 80)
Polyoxyethylene-polyoxypropylene block copolymer (Pluronic F68)	Sodium deoxycholate (NDC)
Polyoxyethylene-polyoxypropylene block copolymer (Pluronic F127)	D-α-Tocopherol polyethylene glycol 1000 succinate (TPGS)
Polyvinyl alcohol (PVA)	Dioctyl sulfosuccinate sodium salt (AOT)
Polyvinyl alcohol-polyethylene glycol copolymer and polyvinyl alcohol (Kollicoat Protect)	Polyethylene glycol (15)-hydroxystearate (Solutol)
Hydroxypropyl methylcellulose (HPMC)	

Supplementary Table 3. Reproducibility of selected SSPN synthesis (varying formulations) with drug loading ≥ 50 wt% (n=3)

Pro Drug	Drug Loading (wt%)	Polymer	Surfactant	Z-average nm (+/-SD)	PDI (+/- SD)
**8**	70	F127	TPGS	267 (26)	0.314 (0.022)
**4**	50	F127	TPGS	702 (111)	0.305 (0.090)
**4**	50	PVA	Tween 20	782 (199)	0.450 (0.152)
**6**	50	F127	TPGS	239 (50)	0.323 (0.042)
**6**	50	PVA	Tween 20	298 (21)	0.339 (0.038)
**6**	50	HPMC	NDC	261 (44)	0.295 (0.019)
**6**	50	HPMC	AOT	293 (9)	0.294 (0.028)
**8**	50	F127	TPGS	196 (6)	0.345 (0.027)
**8**	50	PVA	Tween 20	261 (17)	0.376 (0.038)
**8**	50	HPMC	NDC	217 (10)	0.306 (0.022)
**8**	50	HPMC	AOT	237 (5)	0.296 (0.021)

Supplementary Table 4. Table showing the key parameters used in simulating the systemic distribution of carbonate/carbamate prodrugs, carbamate intermediary and FTC following simulated IM injection.

	FTC	C4 carbamate carbonate	C4 carbamate	C8 carbamate carbonate	C8 carbamate
Molecular Weight	247.45	447.49	347.37	559.69	403.47
Hydrolysis liver (h^{-1})		0.5786	0.0125	0.0694	0.0282
Hydrolysis muscle (h^{-1})		0.0290	0.001	0.0067	0.004
Hydrolysis plasma (h^{-1})		1.4	16.0	8.4	11.1
Plasma Cl (L/h)	19.35	19.35	19.35	19.35	19.35
Release Rate (h^{-1})		0.0015		0.0015	
Liver to plasma ratio	0.8	6.1	1.0	5.5	5.4
Muscle to plasma ratio	0.8	3.2	0.9	3.3	3.4
Tissue (per) to plasma ratio	0.7	8.2	1.1	4.7	4.4

Supplementary Table 5. Pharmacokinetic parameters following oral administration (simulated and observed values) of 400mg FTC at steady state. Values represent mean (minimum and maximum values).

	Simulated	Observed
Cmax (ng/mL)	4666 (3461-6774)	2968 (1582-4165)
Cmin (ng/mL)	57 (1-158)	63 (48-134)
AUC (ng/h/mL)	29052 (27674-29524)	16484 (11524-21388)
IC Cmax (fmol/10^6 cells)	1264 (370-2384)	
IC Cmin (fmol/10^6 cells)	126 (48-206)	

Supplementary Table 6. Simulated pharmacokinetic parameters following: IM injection of SSPN containing C4 prodrug. Values represent mean (± standard deviation)**.**

	FTC	Carbamate carbonate	Carbamate
Cmax (ng/mL)	97 (±21.23)	0.5 (±1.45)	46.5 (±11.90)
Cmin (ng/mL)	34.9 (±7.68)	0.2 (±0.50)	16.3 (±4.26)
AUC (ng/h/mL)	44275.1 (±9754.70)	214.7 (±642.95)	20869.7 (5427.37)
IC Cmax (fmol/10^6 cells)	618.8 (302.48)		
IC Cmin (fmol/10^6 cells)	433.1 (±211.06)		

Supplementary Table 7. Simulated pharmacokinetic parameters following: IM injection of SSPN containing C8 prodrug. Values represent mean (± standard deviation)**.**

	FTC	Carbamate carbonate	Carbamate
Cmax (ng/mL)	146.4 (±19.44)	2.2 (±4.44)	15.3 (±7.44)
Cmin (ng/mL)	52.4 (6.81)	0.8 (1.64)	5.4 (±2.72)
AUC (ng/h/mL)	66643.9 (±8747.28)	988.0 (±2090.69)	6839.5 (±3446.55)
IC Cmax (fmol/10^6 cells)	921.2 (±391.40)		
IC Cmin (fmol/10^6 cells)	644.5 (±270.39)		

Supplementary Table 8. Table showing the key parameters used for the ad hoc model (model 2) in simulating the systemic distribution of carbonate/carbamate prodrugs, carbamate intermediary and FTC following simulated IM injection.

	FTC	C4 carbamate carbonate	C4 carbamate	C8 carbamate carbonate	C8 carbamate
Ka (h^{-1})	0.9				
Molecular Weight	247.45	447.49	347.37	559.69	403.47
Hydrolysis liver (h^{-1})		0.5786	0.0125	0.0694	0.0282
Hydrolysis muscle (h^{-1})		0.0290	0.001	0.0067	0.004
Hydrolysis plasma (h^{-1})		1.4	16.0	8.4	11.1
Plasma Cl (L/h)	24.5	24.5	24.5	24.5	24.5
Release Rate (h^{-1})		0.0015		0.0015	
Liver to plasma ratio	0.8	6.1	1.0	5.5	5.4
Muscle to plasma ratio	0.8	3.2	0.9	3.3	3.4
Tissue (per) to plasma ratio	1.2	8.2	1.1	4.7	4.4

Supplementary Table 9. Pharmacokinetic parameters following oral administration (simulated and observed values) of 400mg FTC at steady state. Observed values represent average values from 3 independent clinical studies. Values represent mean (± standard deviation).

	Simulated	Observed
Cmax (ng/mL)	1857 (± 333.9)	1773 (± 46.1)
Cmin (ng/mL)	36 (± 32.5)	67 (± 25)
AUC (ng/h/mL)	13827 (± 1808.4)	9100 (± 1646)
IC Cmax (fmol/10^6 cells)	3081 (± 1128.9)	
IC Cmin (fmol/10^6 cells)	2676 (± 994.5)	

Supplementary Table 10. Simulated pharmacokinetic parameters following: IM injection of SSPN containing C4 prodrug. Values represent mean (± standard deviation)**.**

	FTC	Carbamate carbonate	Carbamate
Cmax (ng/mL)	75 (± 19.8)	0.2 (± 0.19)	39 (± 10.7)
Cmin (ng/mL)	28 (± 7.8)	0.1 (± 0.07)	15 (± 4.1)
AUC (ng/h/mL)	32949 (± 8856.5)	81 (± 80.1)	17291 (± 4649.1)
IC Cmax (fmol/10^6 cells)	439 (± 176.0)		
IC Cmin (fmol/10^6 cells)	332 (± 132.3)		

Supplementary Table 11. Simulated pharmacokinetic parameters following: IM injection of SSPN containing C8 prodrug. Values represent mean (± standard deviation)**.**

	FTC	Carbamate carbonate	Carbamate
Cmax (ng/mL)	101 (± 26.8)	1 (± 1.8)	23 (± 12.3)
Cmin (ng/mL)	39 (± 10.6)	0.5 (± 0.70)	8 (± 4.7)
AUC (ng/h/mL)	44394 (± 11929.7)	523 (± 798.0)	9882 (± 5352.1)
IC Cmax (fmol/10^6 cells)	608 (± 286.4)		
IC Cmin (fmol/10^6 cells)	444 (± 197.1)		

Supplementary Methods

HPLC method for analysis of prodrug cleavage: Kinetic analysis of prodrug cleavage was performed on a Beckman Gold Nouveau System Gold HPLC using a C_{18} column (Grace Altima, 3 μm C_{18} analytical Rocket® column, 53 mm × 7 mm). All HPLC analyses were performed using the following method: 0% to 100% solvent B over 5 minutes at a flow rate of 3 mL min^{-1} (solvent A: Et_3NHOAc (50 mM, pH 8), solvent B: acetonitrile), which provided adequate separation between the prodrug and observed products. Prodrug concentration was determined by comparison to standard curves acquired at the identified λ_{max} of 305 nm. The identity of product peaks was confirmed by comparison of retention times and UV profiles to those of authentic standards. The products of hydrolysis of prodrugs **1-8** (λ_{max} = 305 nm) are carbamate prodrugs **9-16** (λ_{max} = 305 nm), which reproducibly appear with shorter retention times compared to the corresponding carbonate/carbamate prodrugs. The product of hydrolysis of prodrugs **9-16** is FTC (λ_{max} = 280 nm, retention time = 2.2 min). Representative standard curves and prodrug hydrolysis profiles showing analyte resolution are shown below for conversion of **8** to **16**, and conversion of **16** to FTC. Retention times for other prodrugs are reported in **Table S1**. Unless otherwise noted, all experiments were performed in triplicate. Compounds **8** and **16** were utilized to determine inter- and intra-day accuracy and precision: at the high and middle points of the standard curve these were below 15%; at the low point of the standard curve for both compounds, inter- and intra-day accuracy and precision were within 20% in accordance with convention

Initial rate (human muscle and liver S9) measurements of carbamate cleavage from 9-16 and capecitabine: Reaction mixtures containing mixed gender human skeletal muscle S9 (10.47 mg/mL, Bioreclamation) or phosphate buffer (0.1 M, pH 7.4) and pooled, mixed

gender liver S9 (**9-13**, **15-16** and capecitabine: 10.0 mg/mL; **14**: 5.0 mg/mL) were pre-incubated at 37 °C for 5 min. Reactions were initiated by the addition of prodrugs **9-16** or capecitabine (1 mM + 1% DMSO). After incubation at 37 °C, aliquots were taken at time points measuring initial rate (Muscle S9: CAP, **9-16**: 0.5 min, 1 h, 3 h, 5 h, 24 h, 48 h, 72 h) (Liver S9: **9**: 0.5 min, 1 h, 2 h, 3 h, 4 h, 5 h, 6 h; **10-11**: 0.5 min, 20 min, 40 min, 60 min, 80 min, 100 min, 120 min; **12-13**: 0.5 min, 10 min, 20 min, 30 min, 40 min, 50 min, 60 min; CAP and **14-16**: 0.5 min, 5 min, 10 min, 15 min, 20 min, 25 min, 30 min). These aliquots were quenched in two volumes of ice-cold methanol. The quenched aliquots were then centrifuged at 16873xg for 5 minutes. The supernatant was diluted 10-fold into phosphate buffer (0.1 M, pH 7.4) and analyzed by the HPLC method described above monitoring product depletion at 305 nm.

Half-life (human plasma) measurements of carbamate cleavage from 9-16 and capecitabine: Reaction mixtures containing pooled mixed gender human plasma (Bioreclamation) were pre-incubated at 37 °C for 5 min. Reactions were initiated by the addition of prodrugs **9-16** or capecitabine (1 mM + 1% DMSO). After incubation at 37 °C, aliquots were taken at the following time points: 0.5 min, 1 h, 3 h, 5 h, 24 h, 48 h, 72 h. These aliquots were quenched in two volumes of ice-cold methanol. The quenched aliquots were then centrifuged at 16873xg for 5 minutes. The supernatant was diluted 10-fold into phosphate buffer (0.1 M, pH 7.4) and analyzed by the HPLC method described above monitoring product depletion at 305 nm.

Analysis of prodrug stability during SSPN manufacture: SSPNs were dissolved in DMSO to yield a prodrug concentration of 100 mM based on the prodrug mass per sample. Samples were diluted further to 1 mM in DMSO. These stocks were then diluted 10-fold in phosphate buffer (0.1 M, pH 7.4), giving a final prodrug concentration of 100 μM, prior to HPLC

analysis using the following method: 0% to 100% B over 5 minutes at a flow rate of 3 mL min^{-1} (solvent A: Et_3NHOAc (50 mM, pH 8), solvent B: acetonitrile). SSPN samples were compared to two controls: 1) an excipient control comprised of equal masses of excipients found in SSPNs carried through the same volume and dilution scheme as test samples above, and 2) a prodrug control comprised of unformulated prodrug at the same concentration as prodrug found in SSPNs.

Synthesis of semi-solid prodrug nanoparticles via emulsion templated freeze drying with 50 wt% loading of prodrug: Into separate new 14 mL glass sample vial polymers were weighed out and dissolved to a final concentration of 13.3 mg/mL, whilst surfactants were weighed out and dissolved to a final concentration of 10 mg/mL, both in distilled water. These solutions were left overnight on a rolling mixer to ensure thorough dissolution. Immediately before synthesis of SSPNs, prodrug was removed from the freezer and weighed out in to a fresh 14 mL glass vial. Pro drug was dissolved to a final concentration of 50 mg/mL in chloroform and left on a rolling mixer for 10 minutes to ensure thorough dissolution. Prodrug was not left rolling in solution for any excess time to prevent hydrolysis. For each SSPN sample, 100 µL of surfactant, 300 µL of polymer, and 100 µL of prodrug was added to a 4 mL glass sample vial. This was repeated for all 42 combinations of polymer and surfactant (7x 6) and each prodrug. The final composition yielded SSPNs consisting of 5 mg prodrug (50 wt%), 1 mg surfactant (10 wt%) and 4 mg polymer (40 wt%). The remainder of the synthesis process was the same as for SSPNs at 10 wt% loading.

Determination of SSPN candidate formulations: All samples were dispersed in distilled water to a final concentration of 1 mg/mL of total prodrug mass. Physical characterisation of the particles (after dispersion in water) in terms of hydrodynamic diameter and polydispersity index (PDI) was conducted using DLS Specifically, the following parameters were used:

Particle Type: Nanoparticles (Refractive Index 1.330, Absorption 0.010)

Dispersant: Water (Viscosity 0.8872 cP, Refractive Index 1.330)

Temperature: 25°C

Cell Type: Polystyrene disposable cuvette

Measurement Angle: 172° Back Scatter

Number of Measurements: 3

Number of runs per measurement: Automatic

Candidates were selected based on how easily the dried particle monoliths dispersed in water (at 1 mg/mL), as well as having Z-average hydrodynamic diameters of less than 700 nm, and PDI values of less than 0.4.

IVIVE Model Equations and Parameters: The 7-compartment model was generated using equations from the physB model to simulate organ size, volumes and flow rates (Supplementary Figure 45).[1] The model generates simulated patients based on anthropometric measures and allometric scaling. The volume of distribution was simulated using the Poulin and Theil equation.[2] This method describes the tissue-to-plasma ratio based on the individual organ volumes generated from the physB equations. The values used in the model are shown in Supplementary Table 4 and the key equations are described below:

$$\frac{\Delta FTC_{oral}}{\Delta t} = Ka * Dose \qquad (1)$$

Equation 1 describes the rate of absorption of FTC following an oral dose where Dose is equal to the amount of FTC remaining in the oral compartment and Ka is the absorption constant.

$$\frac{\Delta Drug_{inj}}{\Delta t} = Kim * Dose \quad (2)$$

Equation 2 describes the rate of release of carbonate carbamate prodrug following an IM dose where Dose is equal to the amount of carbonate carbamate prodrug remaining in the injection site and Kim is the release rate.

$$\frac{\Delta Drug_{inj}}{\Delta t} = Qmu * \left(\frac{IM_{area}}{Muscle_{vol}}\right) * \left(\frac{IM_{drug}}{IM_{area}}\right) \quad (3)$$

Equation 3 describes the rate of release of carbonate carbamate prodrug, carbamate intermediate or FTC following an IM dose where Qmu is equal to the flow rate of blood to the injection site. Also represented are the area of the injection site (IMarea), muscle volume and amount of drug available for release.

$$\frac{\Delta Drug_{Blood}}{\Delta t} = Cl * Blood_{drug} * R \quad (4)$$

Equations 4 describes the clearance of carbonate carbamate prodrug, carbamate intermediate or FTC from the blood accounting for the rate clearance (Cl), the blood to plasma ratio (R) and the concentration of drug in the blood.

$$\frac{\Delta Drug_{Li}}{\Delta t} = Qhv * Blood_{drug} - Qhv * Liver_{drug} * \left(\frac{R}{L{:}P}\right) \quad (5)$$

$$\frac{\Delta Drug_{Mu}}{\Delta t} = Qmu * Blood_{drug} - Qmu * Muscle_{drug} * \left(\frac{R}{M{:}P}\right) \quad (6)$$

$$\frac{\Delta Drug_{Ti}}{\Delta t} = Qti * Blood_{drug} - Qti * Tissue_{drug} * \left(\frac{R}{T{:}P}\right) \quad (7)$$

Equations 5, 6 and 7 describes the transfer of carbonate carbamate prodrug, carbamate intermediate or FTC to and from liver (Li), muscle (Mu) and peripheral tissues (Ti) where Qhv, Qmu and Qti are equal to the flow rate of blood to the liver, muscle and peripheral tissues respectively. Also represented are the concentration of each drug in the respective compartments, blood to plasma ratio and the tissue to plasma ratio for liver (L:P), muscle M:P) and peripheral tissues (T:P).

$$\frac{\Delta Drug_{Ti}}{\Delta t} = Hyd_1 * PD_1 \quad (8)$$

$$\frac{\Delta Drug_{Ti}}{\Delta t} = Hyd_2 * PD_2 \quad (9)$$

Equations 8 and 9 describe the hydrolysis (Hyd1) of carbonate carbamate prodrug (PD1) to carbamate intermediate (PD2) and hydrolysis (Hyd2) of carbamate intermediate to FTC. The rates of hydrolysis are specific to each compartment (plasma, liver or muscle and injection site) and where determined experimentally.

$$K_{ic} = \frac{V_{max} * Blood_{drug}}{Km + Blood_{drug}} \quad (10)$$

$$K_{el} = K_{out} * FTC_{TP} \quad (11)$$

Equation 10 describes the anabolism of FTC to form FTC-TP considering he maximum rate of uptake (Vmax), the rate of anabolism (Km) and the concentration of FTC in the blood. Equation 11 shows the rate of elimination of intracellular FTC-TP which is determined by the elimination rate constant (Kout).

Simulation design: A virtual cohort of 50 patients was generated, and a simulated once-daily dose of FTC (400 mg) was validated against clinical data at steady state. The validated model

was then used for LA simulations, IM dose of 2g SSPN over 28 days. Patient age (minimum of 18 years and maximum of 60 years), weight (minimum of 40 kg and maximum of 100 kg), height (minimum of 1.5 m and maximum of 2.1 m), and body mass index (minimum of 18 and maximum of 30) were generated from random normally distributed values.

Details of ad hoc IVIVE modelling: In order to explore the variability in patient data the model was validated against 3 clinical studies (all at steady state receiving a once daily dose of 200mg FTC).[3-5] In order to increase the predictive quality of the simulated data, key model parameters were fit to better represent the data (Supplementary Table 8). The simulated data showed acceptable deviation from the observed clinical data. The C_{max}, C_{min} and AUC varied by +4%, -53% and +52% respectively (Supplementary Table 9). Simulated FTC plasma concentrations fell below the concentration inhibiting 90% of viral replication (IC_{90}, 50 ng/mL) at 13 and 22 days for **4** and **8**, respectively. Simulated intracellular FTC-TP (Supplementary Tables 10 & 11) C_{min} at day 28 maintained simulated intracellular concentrations above the intracellular IC_{50}, 332 (± 132.3) fmol/10^6 cells and 444 (± 197.1) fmol/10^6 cells. The simulated data indicate that even when the model is fit to a range of clinical data, SSPNs of prodrugs **4** and **8** have the potential to sustain concentrations of FTC above the intracellular IC_{50} for 28 days following a single 2 g IM injection.

Supplementary References

1. Bosgra S, van Eijkeren J, Bos P, Zeilmaker M, Slob W. An improved model to predict physiologically based model parameters and their inter-individual variability from anthropometry. *Crit. Rev. Toxicol.,* 2012, **42**, 751–767
2. Poulin P, Theil FP. Prediction of pharmacokinetics prior to in vivo studies. 1. Mechanism-based prediction of volume of distribution. *J. Pharm. Sci.,* 2002. **91**,129 – 156.
3. Molina J-M, Peytavin G, Perusat S, Lascoux-Combes C, Sereni D, RozenbaumW, Chene G. Pharmacokinetics of emtricitabine, didanosine and efavirenz administered once-daily for the treatment of HIV-infected adults (pharmacokinetic sub-study of the ANRS 091 trial). *HIV Med.,* 2004, **5**, 99-104.
4. Wang LH, Begley J, St Claire RL 3rd, Harris J, Wakeford C, Rousseau FS. Pharmacokinetic and pharmacodynamic characteristics of emtricitabine support its once daily dosing for the treatment of HIV infection. *AIDS Res Hum Retroviruses,* 2004, 20, 1173-82.
5. https://liverpool-hiv-hep.s3.amazonaws.com/prescribing_resources/pdfs/000/000/063/original/HIV_FactSheet_FTC_2016_Mar.pdf?1520612387 accessed on 26th November 2018
